# Melatonin‐engineered MSCs‐exosomes deliver USP4 to stabilise ARNTL and inhibit clock rhythmic ferroptosis for enhanced flap survival

**DOI:** 10.1002/ctm2.70565

**Published:** 2026-01-02

**Authors:** Xiaoqiong Jiang, Yu Wang, Xuanlong Zhang, Huiming Deng, Liangyu Fang, Chaire Tafadzwa, Jiangnan Yao, Hao Chen, Anqi Ye, Kailiang Zhou, Xiangwei Ling, Jian Xiao

**Affiliations:** ^1^ Department of Wound Healing The First Affiliated Hospital of Wenzhou Medical University Wenzhou China; ^2^ Oujiang Laboratory (Zhejiang Lab for Regenerative Medicine, Vision and Brain Health), School of Pharmaceutical Sciences Wenzhou Medical University Wenzhou China; ^3^ School of Nursing Wenzhou Medical University Wenzhou China; ^4^ Department of Orthopaedics The Second Affiliated Hospital and Yuying Children's Hospital of Wenzhou Medical University Wenzhou Zhejiang China

**Keywords:** circadian rhythms, exosomes, ferroptosis, flap necrosis

## Abstract

**Background:**

This study investigates the impact of sleep restriction (SR) on flap viability and its underlying mechanisms. It reveals that SR triggers clock rhythmic ferroptosis, which leads to impaired skin barrier function and increased flap necrosis.

**Methods:**

A retrospective analysis of sleep quality in 344 patients undergoing flap surgery proved that SR is a risk factor for flap necrosis. Further research demonstrated that SR increases the level of ferroptosis, disrupts the circadian rhythm of ferroptosis and exacerbates flap damage in human and murine models.

**Results:**

In order to address this clinical issue, the use of melatonin (MT)‐preconditioned bone marrow mesenchymal stromal cells‐derived exosomes (MEXOs) was found to enhance the therapeutic efficacy of flap repair by mitigating clock rhythmic ferroptosis. Mechanistically, MT increased m6A modification to stabilise and enhance the translation of ubiquitin‐specific protease 4 (USP4) mRNA within MEXOs. USP4 delivered by MEXOs directly interacted with and deubiquitinated ARNTL, a core circadian regulator, stabilising its protein levels and suppressing ferroptosis in flap.

**Conclusions:**

These findings identify SR‐induced clock rhythmic ferroptosis as a critical pathological driver of flap failure and propose a novel exosome‐based strategy targeting the USP4–ARNTL axis to enhance skin barrier integrity and flap survival, offering translational potential for clinical reconstructive surgery.

**Key points:**

1. This study identifies SR‐induced clock rhythmic ferroptosis as a pivotal pathological process in flap necrosis.
2. We reveal a potential therapeutic mechanism in which USP4‐enriched MEXOs can effectively repair SR‐induced flap necrosis.
3. USP4‐enriched MEXOs represent a novel therapy for SR‐induced flap necrosis by stabilizing ARNTL to inhibit clock rhythmic ferroptosis.

## Graphical abstract

• Sleep restriction induces clock rhythmic ferroptosis, leading to skin flap necrosis.

• MEXOs deliver USP4 to stabilise ARNTL, restoring circadian rhythm and inhibiting ferroptosis.

• The METTL3/YTHDF1 m6A axis up‐regulates USP4 in melatonin‐preconditioned BMSCs.

• MEXOs represent a novel cell‐free therapy for enhancing flap survival under circadian disruption.

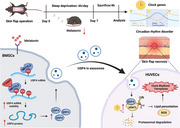

## 1 INTRODUCTION

The skin operates under circadian rhythms regulating transepidermal water loss, permeability, keratinocyte proliferation, blood flow and temperature.^1^ Disruption of these rhythms, such as via sleep restriction (SR)—defined as inadequate sleep duration/quality for physiological needs^2^—impairs skin homeostasis, reducing hydration, elasticity, barrier function and appearance.^3^ In plastic surgery, random‐pattern skin flaps address defects (e.g., ulcers, burns) but face necrosis risks when length‐to‐width ratios exceed 2:1 due to inconsistent blood supply.^4^ Pathological changes like free radical production and inflammatory cell infiltration, exacerbated by circadian disruptions, heighten necrosis susceptibility.^5^ Clinical pre‐tests indicate SR patients are prone to flap necrosis. Postoperatively, therapeutic interventions and pain further compromise sleep, worsening repair outcomes. Addressing SR's impact is critical to optimising flap viability and recovery.^6^ Strategies to mitigate SR effects, such as sleep hygiene interventions or circadian rhythm stabilisation, should be integrated into perioperative care to enhance surgical success and patient outcomes. This highlights the bidirectional link between sleep health and flap repair mechanisms, emphasising SR as a modifiable risk factor in reconstructive surgery.

A number of randomised clinical trials are now investigating the impact of melatonin (MT) on the sleep quality of clinical patients.^7^ *^,^* ^8^ These studies suggest that oral MT improves sleep quality in clinical patients, but highlight the lack of improvement in postoperative patients.^9^, ^10^, ^11^ MT, a regulator of circadian rhythms and sleep, is synthesised in the skin and maintains its homeostasis via receptor pathways.^1^, ^12^ Beyond circadian regulation, MT acts as a potent antioxidant, reducing oxidative stress, inflammation, apoptosis and aging, while enhancing wound healing, preventing photoaging and aiding therapies for dermatitis and psoriasis.^13^ These properties make MT promising for improving skin flap outcomes, especially under SR. However, as oral MT yields limited sleep quality improvement and local efficacy in postoperative patients, we explored topical administration via a carrier to address this issue. Exosomes—nanoscale vesicles secreted by cells—facilitate intercellular communication by transferring proteins, lipids and genetic material.^14^, ^15^ Their cargo capacity, biocompatibility and resistance to degradation make them ideal drug‐delivery vehicles.^16^, ^17^, ^18^ Studies show exosomes enhance fibroblast activity and diabetic wound repair via angiogenesis, fibrogenesis and re‐epithelisation.^19^ While MT‐preconditioned exosomes (MEXOs) may boost skin flap recovery post‐SR, it remains unclear if their efficacy surpasses unmodified exosomes. Additionally, whether benefits arise from exosome genetic cargo or other factors (e.g., MT's direct effects) requires investigation.

Emerging research suggests that SR may disrupt circadian rhythms, thereby predisposing cells to death.^20^, ^21^, ^22^ This form of cell death influences the expression of genes or proteins associated with the circadian clock, and its involvement has been substantiated in conditions like myocardial injury, kidney stones and liver damage.^23^, ^24^, ^25^ Furthermore, SR elevates the expression of genes linked to oxidative stress and immune responses,^26^, ^27^ reinforcing the potential connection between SR and cellular damage. Ferroptosis is a programmed cell death distinct from apoptosis, necrosis and autophagy.^28^ It is initiated by reactive oxygen species (ROS) accumulation from iron‐dependent lipid peroxidation and characterised by GSH depletion, GPX4 inactivation and resultant lipid peroxide buildup.^29^, ^30^ Ferroptosis has been implicated in a variety of pathologies, including neurodegenerative diseases, cancer, myocardial injury and fibrosis, and is increasingly recognised for its potential role in skin damage.^31^ It is hypothesised that SR may induce flap circadian rhythms disorder and activated ferroptosis, leading to the necrosis of flap, as has been proven in other diseases.^32^, ^33^

The study revealed that SR induces clock rhythmic ferroptosis in flap, which is characterised by elevated levels of ferroptosis and disturbances in circadian fluctuations. MEXOs derived from MT‐preconditioned bone marrow mesenchymal stromal cells (BMSCs) exhibited a notable superiority in maintaining skin barrier function and promoting flap survival by mitigating clock rhythmic ferroptosis, compared with those derived from non‐preconditioned BMSCs (EXOs). This enhancement is attributed to the role of MT in increasing m6A modification, which bolsters the translation and stability of mRNA encoding ubiquitin‐specific protease 4 (USP4) within MEXOs. Further mechanistic insights reveal that USP4 transferred by MEXOs directly interacts with and deubiquitinates the clock gene ARNTL in flap, stabilising this core circadian regulator and thus inhibiting clock rhythmic ferroptosis. This suggests that leveraging the anti‐clock rhythmic ferroptosis properties of USP4‐loaded MEXOs could provide a novel approach to treating flap necrosis.

## 2 RESULTS

### 2.1 SR is a risk factor for skin flap necrosis in the clinic

**FIGURE 1:**
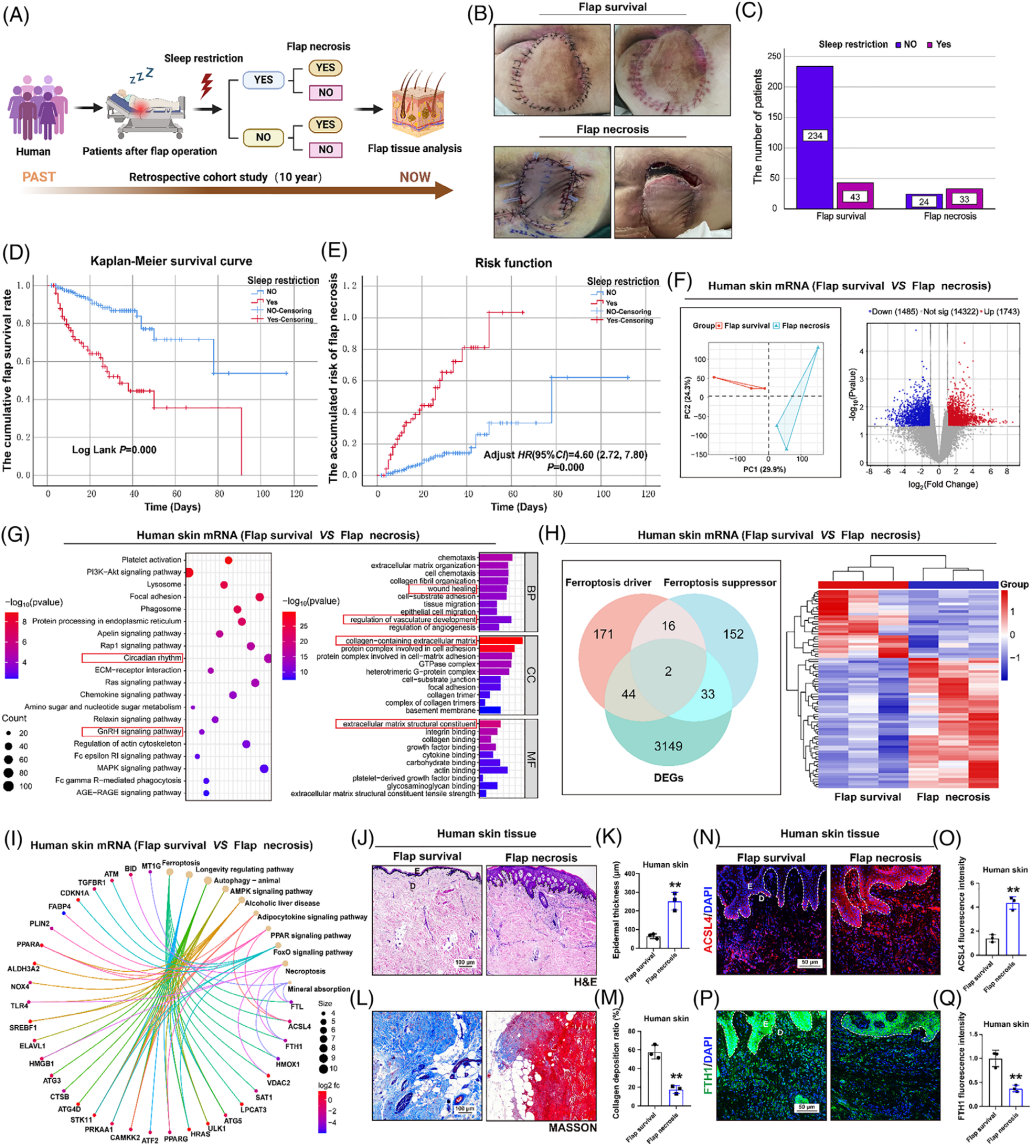
Sleep restriction is a risk factor for skin flap necrosis in the clinic. (A) Follow‐up flowchart for retrospective cohort studies (*n* = 344 patients). (B) Representative visible light images of flap survival and necrosis after flap surgery. (C) Percentage of patients with different flap repair outcomes with SR. (D) Kaplan–Meier survival curve analysis of SR on flap necrosis. (E) Risk function of SR on flap necrosis. (F) The volcano map of DEGs of flap survival and necrosis (*n* = 3 patients/group). (G) A KEGG pathway analysis and GO enrichment analysis were conducted on DEGs. (H) The Venn diagram and heatmap analysis of ferroptosis‐related DEGs. (I) The pathway‐cnet plot of ferroptosis‐related DEGs. (J and K) Representative HE images of patient skin tissue, the skin and epidermal thickness was determined (*n* = 3 patients/group, scale bars = 100 µm). (L and M) Representative Masson's trichrome images, the quantification of the collagen deposition (*n* = 3 patients/group, scale bar = 100 µm). (N–Q) Representative IF images showing ACSL4 (red) and FTH1 (green) fluorescence intensity expression in flap survival and necrosis skin tissue (DAPI staining of the nuclei). Densitometric quantification on the right (*n* = 3 patients/group, scale bars = 50 µm). Student's *t*‐test was performed to compare two groups; bar graph shows the mean ± SEM; *p < .05, **p < .01 and ***p < .001, ns = not significant.

A retrospective analysis (Figure 1A,B) of flap surgery outcomes in patients with pressure injuries at a hospital in south‐west China from 2014 to 2024 (*n* = 344 patients) revealed a postoperative flap necrosis incidence of 16.57% based on an electronic medical record system (Table 1). Then, a correlation analysis was conducted between risk factors associated with flap necrosis, revealing a positive correlation between SR and flap necrosis (*r* = .380, *p *= .000) (Table 2). SR emerged as a significant risk factor of postoperative flap necrosis (Figure 1C). Compared with other risk factors, SR as a predictor of flap necrosis demonstrated a satisfactory predictive efficacy, as evidenced by an area under the curve (AUC) value of  .71 (Table 3). The Kaplan–Meier survival curves further substantiated that the presence of SR in postoperative patients undergoing flap surgery during their hospital stay resulted in a substantial escalation in the incidence of flap necrosis (Figure 1D,E). Finally, a COX proportional hazards regression model was used to adjust for other potential confounding factors associated with flap necrosis, including sedative use, haemodialysis, mechanical ventilation, height, cholesterol, haemoglobin, albumin, FiO_2_, Apache II and hospitalisation time (Table 4). This model further substantiated that SR amplifies the risk of flap necrosis by a factor of 4.60 (HR = 4.60, 95% CI = 2.72, 7.80).

**TABLE 1:** Demographic characteristics and clinical baseline data of skin flap patients.

Variables	Flap survival (*n* = 277)	Flap necrosis (*n* = 57)	*p* Value
Gender, %			.771
Male	93(33.6)	18(31.6)	
Female	184(66.4)	39(68.4)	
Smoking, %			.657
No	156(56.3)	34(59.6)	
Yes	94(33.9)	18(31.6)	
Quitted	27(9.7)	5(8.8)	
Drinking, %			.769
NO	147(53.1)	31(54.4)	
Sometimes	42(15.2)	9(15.8)	
Yes	63(22.7)	10(17.5)	
Quitted	25(9.0)	7(12.3)	
Paralysis, %	79(28.5%)	23(40.4%)	.058
Hypertension, %	151(54.5)	27(47.4)	.325
Diabetes, %	91(32.9)	18(31.6)	.852
Sleep restriction, %	43(15.3)	33(57.9)	.000
Infections, %	134(48.4)	29(50.9)	.731
Vasoactive drugs, %	224(80.9)	45(78.9)	.739
Sedative, %	179(64.6)	23(40.4)	.001
Prednisone, %	97(35.0)	17(29.8)	.451
Diuretic drug, %	157(56.7)	27(47.4)	.198
Hypoglycemic therapy, %			.635
NO	119(43.0)	27(47.4)	
Hypoglycemic drugs	12(4.3)	4(7.0)	
Insulin	98(35.4)	19(33.3)	
Both	48(17.3)	7(12.3)	
Haemodialysis, %	35(12.6)	27(47.4)	.005
Mechanical ventilation, %	91(32.9)	31(54.4)	.001
Height, median, cm	168.0(160.0,172.0)	165.0(158.0,170.0)	.021
Weight, median, kg	62.0(53.5, 70.0)	64.0(51.5, 70.0)	.985
BMI, median, kg/m^2^	22.0(20.0, 24.2)	22.4(20.0, 25.4)	.247
Age, median, year	65.0(53.0,75.0)	66.0(49.5, 76.5)	.787
SBP, median, mmHg	135.0(121.0, 152.0)	134.0(121,154.0)	.938
DBP, median, mmHg	79.0(67.0, 90.0)	78.0(67.5, 88.0)	.745
MAP, median, mmHg	97.7(86.3, 108.8)	96.3(85.5, 108.0)	.710
Cholesterol, mean, mmol/L	4.0 ± 2.1	4.7 ± 1.8	.009
Triglyceride, median, mmol/L	1.1(.8, 1.8)	1.2(.8, 1.5)	.657
Haemoglobin, median, g/L	120.0(105.0, 135.0)	110.0(96.0, 128.0)	.019
Albumin, mean, g/L	35.2 ± 5.8	32.5 ± 6.4	.003
PO_2_, median, mmHg	98.1(85.5, 114.0)	92.0(83.5, 110.0)	.082
FiO_2_, median, %	.3(.2, .4)	.2(.2, .3)	.007
Apache II, median, score	12.0(7.0, 17.0)	14.0(10.0, 20.0)	.021
NRS, median, score	1.0(.0, 2.0)	1.0(.5, 2.0)	.296
MBI, median, score	25(15, 45)	30(20, 45)	.525
VAS, median, score	2.4 ± 2.3	3.0 ± 2.0	.065
Body temperature, mean, °C	36.9 ± .6	36.8 ± .7	.683
Hospitalisation time, median, day	13(7, 26)	18(11, 28)	.048

Abbreviations: BMI, body mass index; SBP, systolic blood pressure; DBP, diastolic blood pressure; MAP, mean arterial pressure; NRS, nutrition risk screening; MBI, modified Barthel index; PO_2_, oxygen partial pressure; FiO_2_, fraction of inspired O_2_; Apache II, acute physiology and chronic health evaluation II; VAS, visual analogue scale.

**TABLE 2:** Spearman rank correlation between variables and skin flap necrosis.

Variables	*N*	*r*	*p* Value
Sleep restriction	334	.380	.000
Sedative	334	−.187	.001
Haemodialysis	334	.155	.004
Mechanical ventilation	334	.252	.000
Height	334	−.126	.022
Cholesterol	334	.130	.017
Haemoglobin	334	−.116	.035
Albumin	334	−.160	.003
Apache II	334	.13	.017

Abbreviation: Apache II, acute physiology and chronic health evaluation II.

**TABLE 3:** Prediction ability of different variables for skin flap necrosis.

Variables	AUC	AUC (95% CI)	Sensitivity	Specificity	Youden's index	*p* Value
Sleep restriction	.71	.63, .79	57.9	84.5	.424	.000
Sedative	.621	.541, .702	64.6	59.6	.242	.004
Haemodialysis	.563	.488, .639	12.6	100.0	.126	.133
Mechanical ventilation	.668	.594, .741	56.3	77.2	.341	.000
Height	.596	.516, .676	66.4	45.6	.120	.022
Cholesterol	.641	.564, .718	64.9	57.8	.227	.001
Haemoglobin	.598	.518, .769	54.4	63.2	.165	.019
Albumin	.637	.558, .717	56.1	67.1	.232	.001
Apache II	.597	.520, .674	72.6	40.4	.13	.021

Abbreviations: AUC, area under the curve; CI, confidence interval.

**TABLE 4:** Univariate and multivariate COX regression analysis for the skin flap necrosis.

Variables	*N*	Sleep restriction (%)	Unadjusted		Adjusted^a^	
			HR (95% CI)	*p* Value	HR (95% CI)	*p* Value
Flap necrosis				.000		.000
No	277	43(15.3)	1.00		1.00	
Yes	57	33(57.9)	5.16(3.02, 8.83)		4.60 (2.72, 7.80)	

Abbreviations: HR, hazard ratio; CI, confidence interval.

^a^Results were adjusted for sedative, haemodialysis, mechanical ventilation, height, cholesterol, haemoglobin, albumin, FiO_2_, Apache II and hospitalisation time.

### 2.2 Skin flap necrosis activates both the circadian rhythm and the ferroptosis pathway

In order to further explore the molecular mechanisms existing between flap necrosis and SR, skin tissues from flap necrosis and flap survival were subjected to transcriptome analysis. The volcano plot showed that a total of 3228 differentially differentiated genes (DEGs) were detected. Of these, 1743 were up‐regulated genes and 1485 were down‐regulated genes (Figure 1F). Enrichment analysis of these DEGs revealed that they were enriched in ‘wound healing’, ‘regulation of vasculature development’, ‘collagen−containing extracellular matrix’ and ‘extracellular matrix structural constituent’. The KEGG pathway analysis revealed that DEGs were enriched in the ‘circadian rhythm’, followed by the ‘GnRH signalling pathway’. To systematically assess how different forms of cell death—such as apoptosis, necrosis and pyroptosis—affect flap necrosis, we tested corresponding inhibitors in animal models. Inhibition of apoptosis, necrosis, pyroptosis and ferroptosis all led to notable improvements in flap necrosis area, blood flow and skin temperature following SR induction (Figure S1A–F). The most marked improvement, however, was observed after ferroptosis inhibition. A dataset of 419 ferroptosis‐related genes was acquired from the FerrDb database. The intersection between this ferroptosis gene set and our dataset of DEGs was then examined, leading to the identification of 79 ferroptosis‐related DEGs. These were subsequently categorised into 44 up‐regulated and 35 down‐regulated genes (Figure 1H). The cnet‐plot function is used to visualise the association between DEGs and the ferroptosis pathway (Figure 1I). Immunofluorescence (IF) staining further validated the differential expression of the ferroptosis marker gene ACSL4/FTH1 in human flap tissue (Figure 1N–Q). Histological staining demonstrated increased epidermal thickness, decreased collagen deposition during flap necrosis (Figure 1J–M). It is hypothesised, based on the current evidence, that there is an association of flap necrosis with circadian rhythms and ferroptosis.

### 2.3 Characterisation of exosomes and identification of USP4 up‐regulation in MEXOs

**FIGURE 2:**
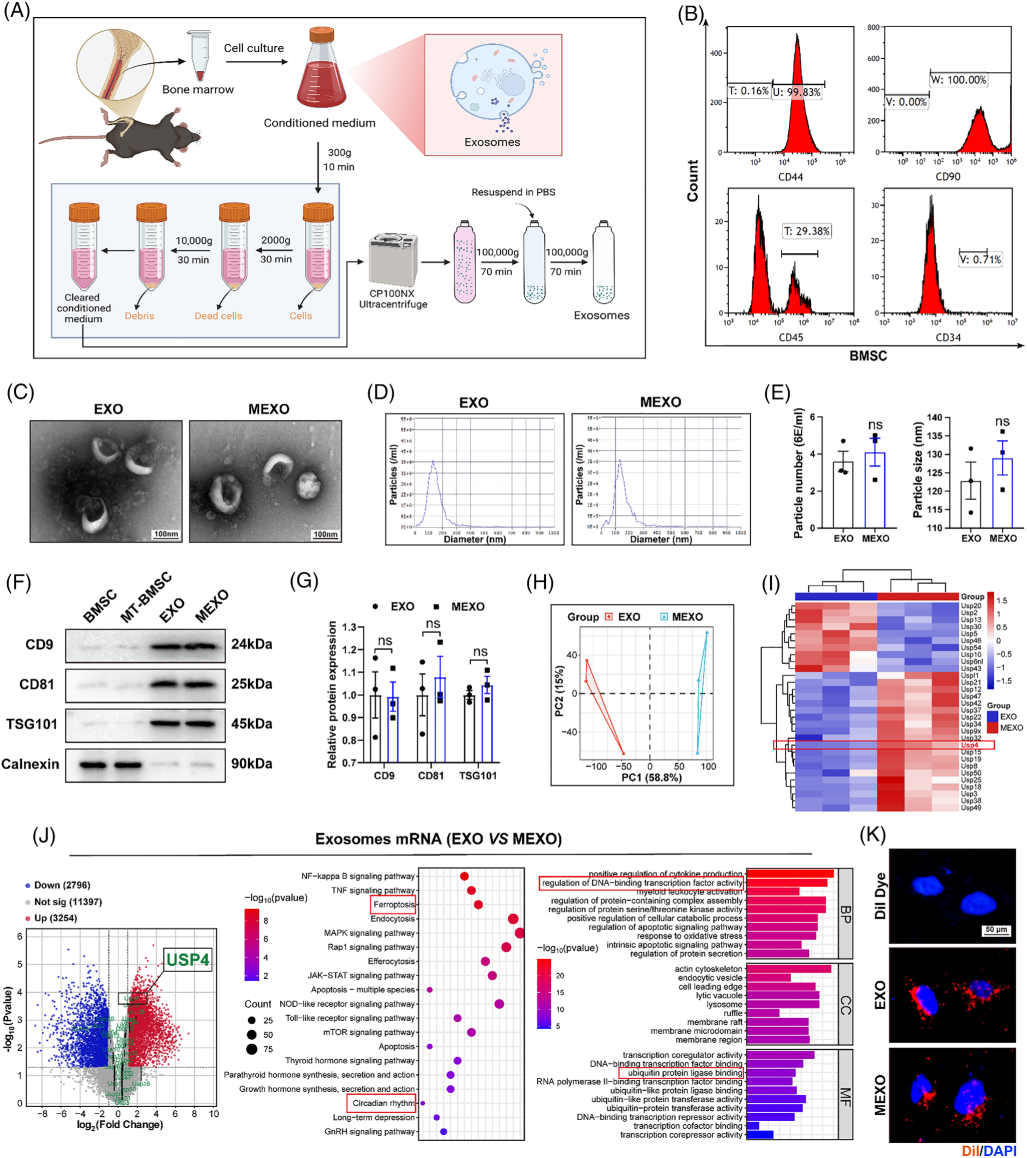
Characterisation of EXOs/MEXOs and identification of USP4 up‐regulation for skin flap repair. (A) BMSCs‐derived exosome extraction process. (B) Identification of BMSCs by flow cytometry (CD90, CD105, CD45 and CD34) (*n* = 3/group). (C) Morphology of EXOs and MEXOs under TEM (*n* = 3/group). (D and E) NTA analysis showing particle distribution of EXOs and MEXOs, mean particle number per mL and particle size of exosomes (*n* = 3/group). (F and G) WB analysis of biomarkers of exosomes, CD9, CD81 and TSG101 and negative control, calnexin (*n* = 3/group). (H) Principal component analysis of EXOs and MEXOs (*n* = 3/group). (I) The heatmap of DUBs of EXOs and MEXOs. (J) The volcano map of DEGs of EXOs and MEXOs (*n* = 3/group). A KEGG pathway analysis and GO enrichment analysis were conducted on DEGs. (K) Uptake of red fluorescent dye Dil‐labelled exosomes into HUVECs (*n* = 3/group, scale bar = 50 µm). Student's *t*‐test was performed to compare two groups; bar graph shows the mean ± SEM; *p < .05, **p < .01 and ***p < .001, ns = not significant.

MT has been identified as a core circadian rhythm‐regulating hormone and possesses antioxidant properties. In addition, the cargo capacity, biocompatibility and resistance of EXOs to degradation render them ideal drug‐delivery vehicles. The present study investigates the characteristics of EXOs secreted by MT‐pretreated BMSCs (MEXOs) and compares them with basic EXOs, in order to assess their therapeutic potential for skin flap necrosis associated with circadian rhythm disorders and ferroptosis. BMSCs were phenotypically characterised using flow cytometry and identified as positive for the surface markers CD90 and CD44, while being negative for haematopoietic markers CD45 and CD34 (Figure 2A,B). According to transmission electron microscopy (TEM) images, EXOs and MEXOs both displayed a characteristic cup‐shaped or spherical morphology, around 100 nm in diameter (Figure 2C). The consistency in exosome preparation was further affirmed by nanoparticle tracking analysis, which indicated analogous size distribution profiles between the two groups (Figure 2D). Additionally, no significant differences were observed in the mean nanoparticle size or concentration (Figure 2E). Western blot (WB) analysis was utilised to assess the protein composition of the exosomes, revealing the presence of typical exosome surface markers such as CD9, CD81 and TSG101. Notably, the negative exosome marker calnexin was absent in both types of exosomes, with no observable differences between EXOs and MEXOs (Figure 2F,G). This study confirmed that the isolated exosomes met the expected standards for morphology, size, concentration and surface markers, establishing the foundation for subsequent functional studies.

With the fundamental properties of both exosome types validated as comparable, the focus shifted to assessing their differential biological activities and underlying molecular profiles. We found that ‘ferroptosis’, ‘circadian rhythm’ and ‘ubiquitin protein ligase binding’ differed between the EXOs and MEXOs in KEGG and GO analyses using transcriptome analysis (Figure 2J). Furthermore, differential expression of deubiquitinating enzymes (DUBs) was observed, with MT treatment resulting in the up‐regulation of 20 DUBs in MEXOs samples. USP4 exhibited the most significant up‐regulation ((Figure 2I). Combined with the finding in an animal model that inhibition of ferroptosis significantly ameliorates SR leading to a decrease in USP4 in vascular endothelial cells (Figure S1G,H). Subsequent experiments focused on the role of USP4 in regulating flap ferroptosis related to SR. Finally, the exosomes were labelled with Dil dye and co‐cultured with human umbilical vein endothelial cells (HUVECs) for 24 h. The successful uptake of both types of exosomes by the HUVECs when compared with the blank dye group, demonstrating their potential utility in cellular communication and therapeutic applications (Figure 2K).

### 2.4 MEXOs promote skin flap survival and skin barrier function recovery disrupted by SR in mice

**FIGURE 3:**
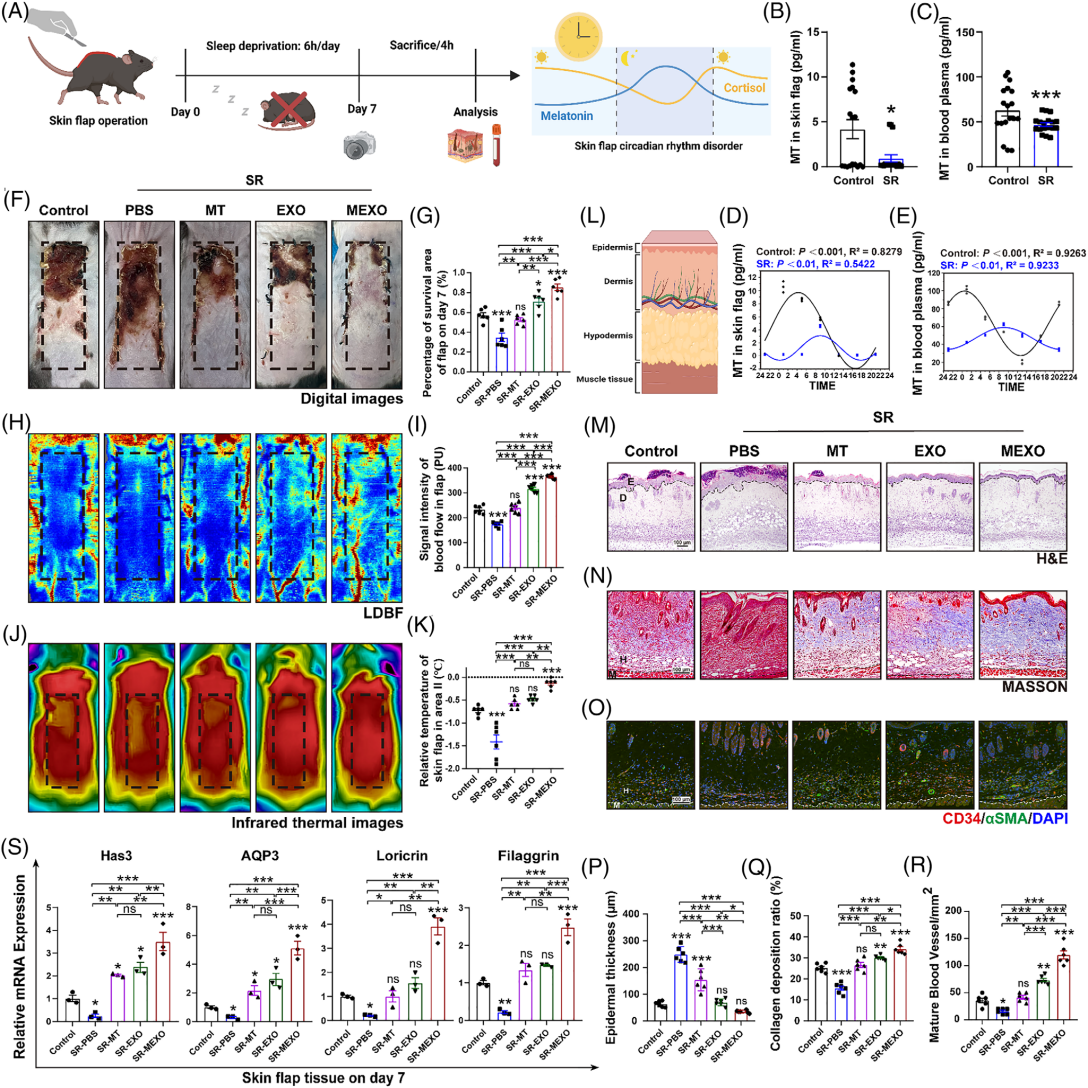
MEXOs promote skin flap survival and skin barrier function recovery disrupted by sleep restriction in mice. (A) Sleep deprivation process after flap surgery in mice. (B and C) Detection of MT changes in flap and plasma on day 7 after SR was measured using an ELISA assay (*n* = 18 mice/group). (D and E) Circadian rhythm patterns for the 24 h cycle of melatonin (*n* = 3 mice/group). *p *< .05 calculated by the Circa Compare algorithm was considered a circadian rhythm. The curve indicates the best fit to the points by cosinor analysis. *R* ^2^ value represents the degree of the fitting. (F and G) The necrotic area was imaged on day 7 after SR, the percentage of survival area was analysed (*n* = 6 mice/group). (H and I) Subcutaneous blood flow was detected by LDBF, the intensity signal of blood flow was analysed (*n* = 6 mice/group). (J and K) Skin temperature was monitored using IRT, the flap temperature was analysed (*n* = 6 mice/group). (L) Anatomy of the flap tissue. (M and P) Representative HE images of flap. The skin and epidermal thickness was determined (*n* = 6 mice/group, scale bar = 100 µm). (N and Q) Representative Masson's trichrome images, the quantification of the collagen deposition (*n* = 6 mice/group, scale bar = 100 µm). (O and R) Representative IF images for CD34 (red) and α‐SMA (green) of flap. The number of CD34 and α‐SMA‐positive blood vessels (mm^2^) were counted and analysed (*n* = 6 mice/group, scale bar = 100 µm). (S) Skin barrier‐related gene expression changes (*n* = 3 mice/group). One‐way ANOVA was used for analysis that involved more than two groups; bar graph shows the mean ± SEM; *p < .05, **p < .01 and ***p < .001, ns = not significant.

A modified McFarlane flap model was utilised on the backs of experimental mice subjected to 1 week of SR (Figure 3A). Considering MT critical function in regulating circadian rhythms and sleep–wake cycles, we performed a rhythmic analysis of MT concentrations in flap and blood plasma post‐SR. The results indicated that mesor (mean) and amplitude of MT were decreased, and the acrophase (peak timing of the rhythm) of MT was delayed in both flap and blood plasma post‐SR (Figure 3B–E). This also demonstrated the success of modelling SR in mice. In addition, changes in MT levels and disturbances to the circadian rhythm adversely affect crucial physiological processes for skin repair. This included influencing the area of flap survival, blood flow establishment and skin barrier function.

Among the treatments evaluated, the group receiving exosomes derived from MEXOs demonstrated a significant enhancement in flap survival compared with groups treated with MT or EXOs alone (Figure 3F,G). Additionally, the treatment showed no signs of acute systemic toxicity (Figure S1I). Laser Doppler imaging showed the highest blood flow in the MEXOs‐treated group (Figure 3H,I). Infrared thermography (IRT), used to monitor skin temperature, indicated a decrease in temperature in the necrotic distal regions following SR. Treatment with MEXOs notably increased skin temperature and reduced the extent of hypothermia (Figure 3J,K). Furthermore, the expression of CD34 and α‐SMA in the micro‐vessels of the flap was elevated post‐MEXO treatment, suggesting enhanced angiogenesis (Figure 3O,R). Histological analysis via haematoxylin and eosin (HE) staining demonstrated a significant reduction in skin and epidermal thickness (Figure 3,P), with an increase in collagen fibre density (Figure 3N,Q). The expression levels of genes involved in hyaluronic acid synthesis and encoding aquaporin, as well as those of genes crucial for the formation of the cutaneous stratum corneum barrier, such as loricrin and filaggrin (Figure 3S), were found to be able to restore the expression levels of these critical barrier‐forming genes. These results illustrate that SR disrupts skin barrier function and impairs angiogenesis, while MEXO treatment not only ameliorates these disruptions but also promotes flap angiogenesis and improves overall skin barrier function.

### 2.5 MEXOs inhibit SR‐induced ferroptosis in skin flap in mice

**FIGURE 4:**
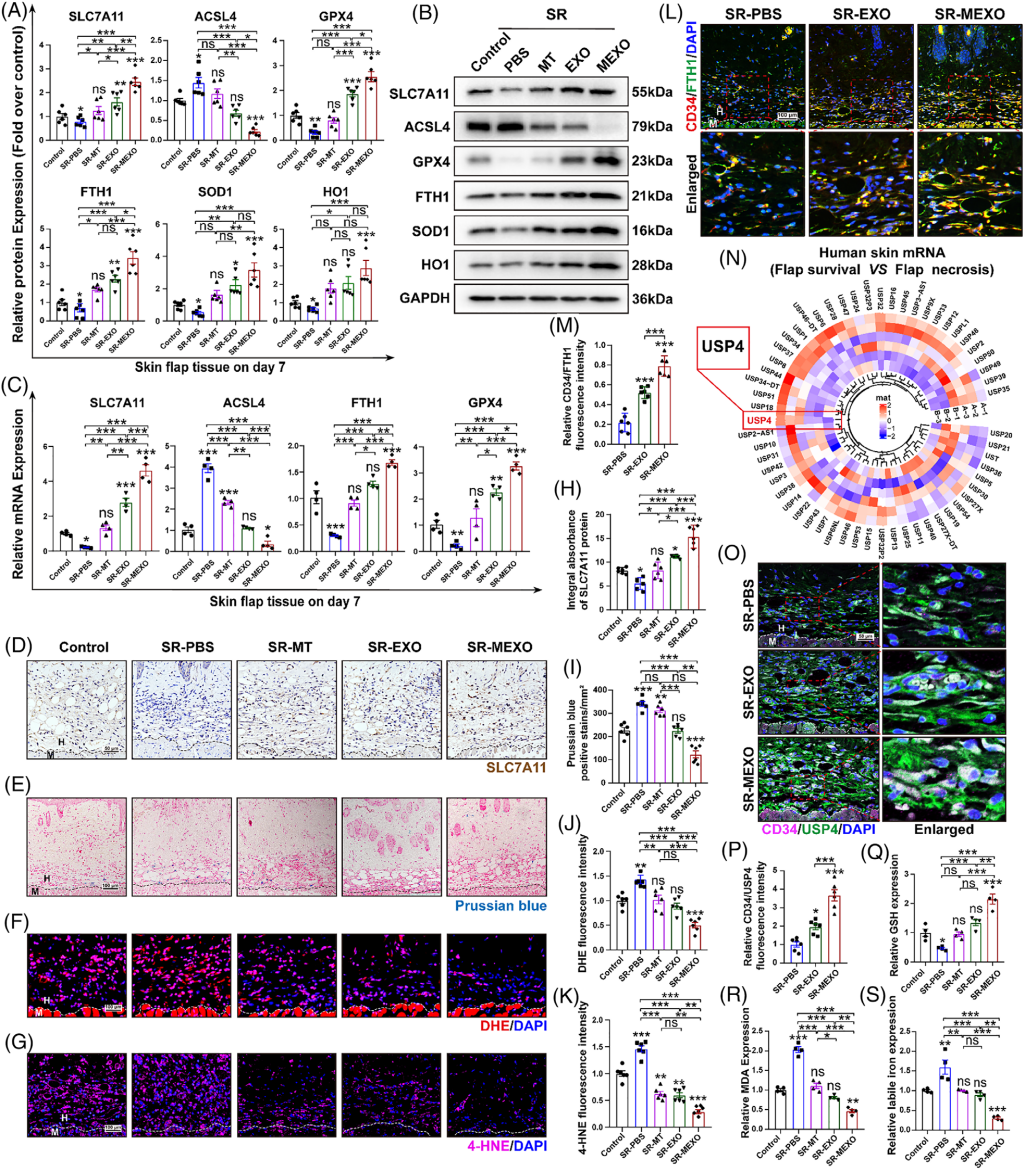
MEXOs inhibit sleep restriction‐induced ferroptosis in skin flap in mice. (A and B) WB was used to detect the expression of ferroptosis‐associated proteins in skin flap on day 7 (*n* = 6 mice/group). (C) qPCR was used to detect the mRNA expression of ferroptosis‐associated proteins (*n* = 6 mice/group). (D and H) The expression of SLC7A11 in skin flap on day 7 was analysed by IHC (*n* = 6 mice/group, scale bar = 50 µm). (E and I) Prussian blue staining was used to detect the iron ion content (*n* = 6 mice/group, scale bar = 100 µm). (F and J) Frozen sections of the skin tissues were stained with DHE (red; a ROS fluorescent probe) (*n* = 6 mice/group, scale bar = 100 µm). (G and K) The expression of 4‐HNE (red) in flap was analysed by IF (*n* = 6 mice/group, scale bar = 100 µm). (L and M) The relative expression of CD34 (red)/FTH1 (green) in flap was analysed by IF (*n* = 6 mice/group, scale bar = 100 µm). (N) The heatmap of specific ubiquitinase families in flap survival and necrosis tissue (*n* = 3 patients/group). (O and P) The relative expression of CD34 (red)/USP4 (green) in flap was analysed by IF (*n* = 6 mice/group, scale bar = 100 µm). (Q–S) The relative values of GSH, MDA and Fe^2+^ concentrations in flap (*n* = 4 mice/group). One‐way ANOVA was used for analysis that involved more than two groups; bar graph shows the mean ± SEM; *p < .05, **p < .01 and ***p < .001, ns = not significant.

In order to address the potential confounding effect of stress induced by the SR procedure, which resulted in flap necrosis, plasma and flap corticosterone levels were measured. While the administration of SR led to a significant elevation in corticosterone levels in comparison with the control group (Figure S2A), the levels remained similarly elevated in the EXOs‐ and MEXOs‐treated SR groups with the exception of the clock rhythmicity recovery (Figure S2J,K). This finding suggests that the therapeutic effects of MEXOs on flap survival are not primarily attributable to stress reduction, but rather to the specific regulation of clock rhythmicity. Additionally, the present study demonstrates that SR significantly induces ferroptosis in mouse flaps, an effect ameliorated by ferroptosis inhibitors (Figure S2A–D). Specifically, WB analysis (Figure 4A,B) and qPCR (Figure 4C) revealed an increase in ACSL4, a marker of ferroptosis and a decrease in the levels of SLC7A11 and GPX4, both of which are key anti‐ferroptosis markers, along with lower levels of FTH1, involved in iron sequestration. This was further demonstrated by labelling vascular endothelial cells with CD34 and co‐localising using the FTH1 ferroptosis marker (Figure 4L,M). The metabolic balance between oxidative and antioxidative processes, crucial for cellular homeostasis, was assessed by measuring the expression of SOD1 and HO1, both of which were found to be significantly decreased following SR. The immunohistochemical results of SLC7A11 were also consistent with the above results (Figure 4D,H). Additionally, high iron content in the tissues was evident from Prussian blue staining (Figure 4E,I). The intensity of dihydroethidium (DHE) and 4‐hydroxynonenal (4‐HNE), markers for oxidative stress and lipid peroxidation, respectively, was found to increase after SR (Figure 4F,J,G,K). The glutathione (GSH), malondialdehyde (MDA) and Fe^2+^ level, markers for ferroptosis, showed a decrease in GSH levels, with concurrent increases in MDA and Fe^2+^ levels in flap after SR (Figure 4Q–S).

Given the potential of MEXOs to specifically regulate circadian rhythms, we examined the expression patterns of ferroptosis‐related genes in skin flaps and observed consistent circadian fluctuations. Specifically, SR was found to disrupt these rhythms, decreasing the mesor and amplitude while delaying the acrophase of SLC7A11, GPX4 and FTH1 (Figure S2L–N). Since SR appears to concurrently exacerbate ferroptosis and induce circadian rhythm disruption, we propose the concept of ‘clock rhythmic ferroptosis’ to describe this phenomenon. Notably, MEXOs treatment not only significantly attenuated ferroptosis but also restored circadian rhythmicity, with a superior efficacy compared with both MT and EXOs treatments.

Based on the above evidence, we hypothesise that MT intervention exerts its therapeutic effect by altering USP4 levels in MEXOs. This mechanism would explain why MEXOs is more efficacious than treatment with EXOs alone or direct MT application. Meanwhile, we also observed significantly reduced USP4 expression in both human necrotic flap tissue (Figures 4N and S2H,I) and a mouse model of SR‐induced flap necrosis (Figure S2F,G), via IF staining co‐localised with the endothelial marker CD34. Notably, MEXOs treatment enhanced USP4 expression in vascular endothelial cells more potently than EXOs (Figure 4O,P). This supports that SR triggers flap ferroptosis by down‐regulating USP4, while MEXOs exert a protective effect by up‐regulating it. MEXOs represent a promising therapy for SR‐induced clock rhythmic ferroptosis, potentially through modulating USP4 in endothelial cells.

### 2.6 MEXOs protect the function of HUVECs and suppress ferroptosis

**FIGURE 5:**
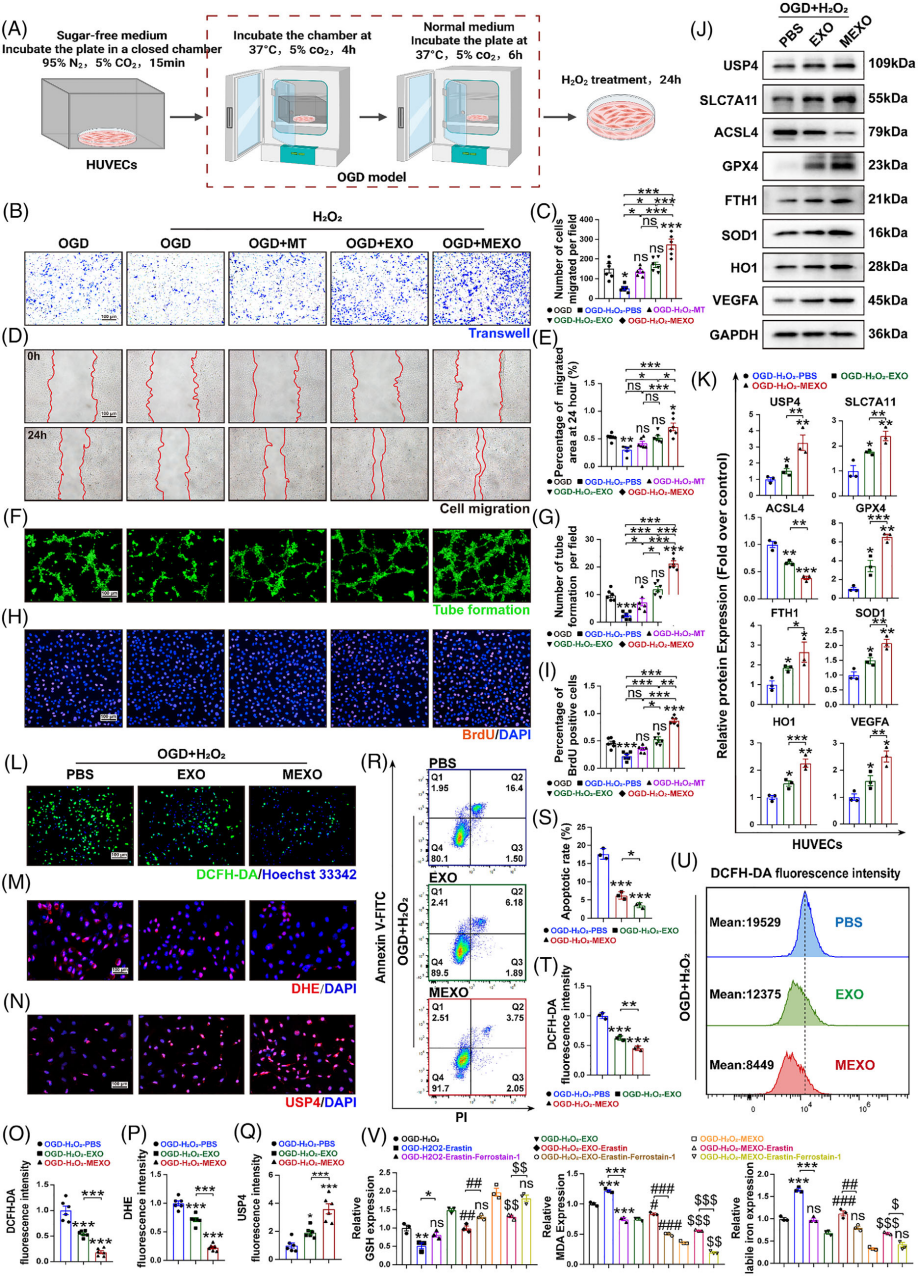
MEXOs protect the function of HUVECs and suppress ferroptosis. (A) Preparation of OGD model and H_2_O_2_ stimulation of HUVECs. (B and C) Cell migration assays were performed on HUVECs after 24 h of different treatments, and the presented results were obtained after 24 h of culture in chambers, quantification and analysis of the number of migrated cells (*n* = 6/group, scale bars = 100 µm). (D and E) Scratching assays were performed on HUVECs, and the presented results were obtained after 0 and 24 h of culture, quantification and analysis of the migrated area of cells (*n* = 6/group, scale bars = 100 µm). (F and G) An in vitro angiogenesis (tube formation) assay was performed on HUVECs, and the presented results were obtained after 4 h of culture (*n* = 6/group, scale bars = 100 µm). (H and I) The EdU assay showed the proliferation of HUVECs (*n* = 6/group, scale bars = 100 µm). (J and K) WB was used to detect the expression of ferroptosis‐associated proteins and USP4 expression in HUVECs (*n* = 3/group). (L and O) Detection and quantification of ROS levels in HUVECs by oxidation‐sensitive fluorescent probe DCFH‐DA (green, *n* = 6/group, scale bars = 100 µm). (M and P) Detection and quantification of ROS levels in HUVECs by DHE staining (red, *n* = 6/group, scale bars = 100 µm). (N and Q) The expression of USP4 (red) in HUVECs was analysed by IF (*n* = 6/group, scale bar = 100 µm). (R–U) The level of apoptosis and ROS production was detected by flow cytometric analysis (*n* = 3/group). (V) The relative values of GSH, MDA and Fe^2+^ concentrations in HUVECs with Erastin and ferrostain‐1 treatment (*n* = 4/group). One‐way ANOVA was used for analysis that involved more than two groups; bar graph shows the mean ± SEM; *p < .05, **p < .01 and ***p < .001, ns = not significant.

To evaluate the protective effects of MEXOs on vascular endothelial cell function, HUVECs were subjected to oxygen glucose deprivation (OGD) and exposed to hydrogen peroxide (H_2_O_2_) to simulate the in vivo conditions of mice (Figure 5A). The viability and functional responses of HUVECs were assessed using a series of in vitro assays, including cell migration, scratch assays and angiogenesis (tube formation) assays. The results demonstrated that HUVECs treated with MEXOs showed a significant increase in cell migration than MT and EXOs (Figure 5B,C). The area covered by migrating cells in scratch assays was notably larger in the MEXOs‐treated group, indicating enhanced wound closure capabilities (Figure 5D,E). Furthermore, angiogenesis assays revealed that MEXOs significantly boosted the angiogenic capacity of HUVECs, leading to more extensive and complex tube formation (Figure 5F,G). Additionally, Brdu staining confirmed that all three treatments—MT, EXOs and MEXOs—enhanced the proliferative capacity of the cells. Notably, MEXOs exhibited the most significant improvement (Figure 5H,I). These findings underscore the therapeutic potential of MEXOs in enhancing endothelial cell function.

The experiments conducted on HUVECs under conditions of OGD and H_2_O_2_ exposure revealed that these stressors significantly intensified ferroptosis events in the cells, as evidenced by WB analysis (Figure 5J,K). Treatment with MEXOs effectively reversed the ferroptosis. This reversal was characterised by a decrease in ACSL4 and increases in SLC7A11, GPX4 and FTH1, all of which are crucial for reducing oxidative stress and inhibiting ferroptosis. This finding is further corroborated by the diminished levels of oxidative stress, as evidenced by DHE staining (Figure 5M,P) and DCFH‐DA probe labelling (Figure 5L,O,T,U). Apoptosis flow cytometry also showed that MEXOs significantly reduced late apoptosis following oxidative stress (Figure 5R,S). Additionally, biochemical assays showed a decrease in GSH levels and increases in MDA and Fe^2+^ levels after ferroptosis induction. These changes were effectively mitigated by treatment with MEXOs, which restored GSH levels and reduced MDA and Fe^2+^ levels (Figure 5V). The results demonstrate that MEXOs have the potential to inhibit HUVECs ferroptosis.

Further analyses focused on the expression of USP4 within the cellular environment, particularly in response to treatment with MEXOs. Our results indicate a significant elevation in the expression of USP4 in the MEXOs‐treated group. This finding was substantiated through WB and corroborated by IF staining (Figure 5N,Q). The observed increase in USP4 suggests a mechanistic pathway through which MEXOs exert their protective effects. Specifically, by elevating USP4 levels, MEXOs protect the function of HUVECs and suppress ferroptosis.

### 2.7 MEXOs inhibit ferroptosis by transferring USP4, promoting the skin flap survival and skin barrier function recovery

**FIGURE 6:**
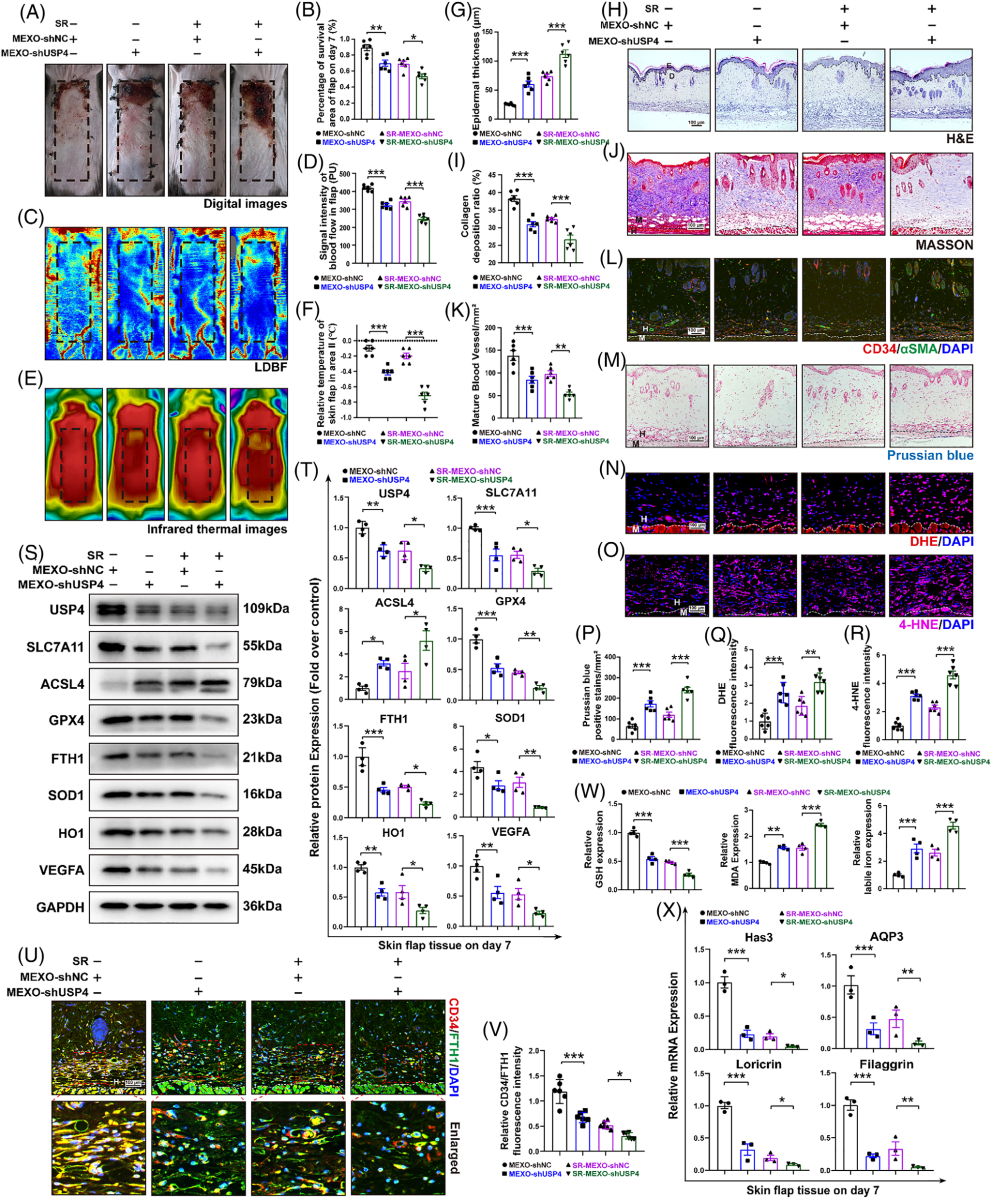
MEXOs promote skin flap survival and skin barrier function recovery after sleep restriction by transferring USP4. (A and B) The necrotic area was imaged on day 7 after SR, and the percentage of survival area was analysed (*n* = 6 mice/group). (C and D) Subcutaneous blood flow was detected by LDBF, and the intensity signal of blood flow was analysed (*n* = 6 mice/group). (E and F) Skin temperature was monitored using IRT, the flap temperature was analysed (*n* = 6 mice/group). (G and H) Representative HE staining of flap in different groups. The skin and epidermal thickness was determined (*n* = 6 mice/group, scale bar = 100 µm). (I and J) Representative Masson's trichrome images, and the quantification of the collagen deposition (*n* = 6 mice/group, scale bar = 100 µm). (K and L) Representative IF images for CD34 (red) and α‐SMA (green) of flap. The number of CD34 and α‐SMA‐positive blood vessels (mm^2^) were counted and analysed (*n* = 6 mice/group, scale bar = 100 µm). (M and P) Prussian blue staining was used to detect the iron ion content (*n* = 6 mice/group, scale bar = 100 µm). (N and Q) Frozen sections of the skin tissues were stained with DHE (red, *n* = 6 mice/group, scale bar = 100 µm). (O and R) The expression of 4‐HNE (red) in flap was analysed by IF (*n* = 6 mice/group, scale bar = 100 µm). (S and T) WB was used to detect the expression of ferroptosis‐associated proteins in flap (*n* = 4 mice/group). (U and V) The relative expression of CD34 (red)/USP4 (green) in flap was analysed by IF (*n* = 6 mice/group, scale bar = 100 µm). (W) The relative values of GSH, MDA and Fe^2+^ concentrations in flap (*n* = 4 mice/group). (X) Skin barrier‐related gene expression changes (*n* = 3 mice/group). One‐way ANOVA was used for analysis that involved more than two groups; bar graph shows the mean ± SEM; *p < .05, **p < .01 and ***p < .001, ns = not significant.

Based on in vitro and in vivo experiments, we confirmed that exosomes derived from USP4‐overexpressing BMSCs (MEXOs [Flag‐USP4]) successfully deliver functional Flag‐USP4 protein into target cells, increase total USP4 levels and facilitate its interaction with endogenous ARNTL (Figure S3A–J). Then, the study generated MEXOs with reduced USP4 levels (MEXOs‐shUSP4). Comparison of flap treated with these modified exosomes and those treated with a control shRNA (MEXOs‐shNC) revealed a significant decrease in USP4 protein expression in the MEXOs‐shUSP4 group, both with and without SR (Figure 6S,T). Treatments with MEXOs‐shUSP4 resulted in poorer survival outcomes compared with MEXOs‐shNC. This was evident from analyses of the necrotic area of the flap, blood flow signals and skin temperature (Figure 6A–F). Furthermore, IF staining indicated that angiogenesis within flap was decreased following treatment with MEXOs‐shUSP4 (Figure 6K,L). The study also explored the structural effects of MEXOs‐shUSP4 on skin and epidermal thickness as well as the arrangement of collagen fibres through HE staining and Masson's trichrome staining (Figure 6G–J). The expression of genes related to skin barrier function was found to decrease after treatment with MEXOs‐shUSP4, further suggesting a diminished recovery in skin barrier function (Figure 6X). These data collectively demonstrate that the ability of MEXOs to promote flap angiogenesis and skin barrier function recovery is dependent on USP4.

Furtherly, WB indicated that MEXOs‐shUSP4 treatment led to heightened ferroptosis activity, evidenced by increased ACSL4 and reduced GPX4 and FTH1 in vivo (Figure 6S,T). It also demonstrated levels of ferroptosis in vascular endothelial cells by using IF double staining (Figure 6U,V). Additionally, Prussian blue staining showed a significant escalation in iron ion release within these tissues (Figure 6M,P). The silencing USP4 markedly decreased the expression of HO1 and SOD1, weakening cellular defences against oxidative stress (Figure 6S,T). The intensities of DHE and 4‐HNE were also found that MEXOs‐shNC more effectively mitigated these markers than MEXOs‐shUSP4 (Figure 6N,O). Quantitative assessments of GSH, MDA and Fe^2+^ revealed decreased GSH levels and increased MDA and Fe^2+^ levels following treatment with MEXOs‐shUSP4 (Figure 6W). These results suggest that MEXOs inhibit ferroptosis, enhancing flap viability and skin barrier function recovery by delivering USP4.

### 2.8 MEXOs inhibit ferroptosis by shuttling USP4, protecting the function of HUVECs

The results revealed a marked decrease in USP4 expression in HUVECs treated with MEXOs‐shUSP4 compared with those treated with MEXOs‐shNC. This reduction was further exacerbated following treatment with the Erastin and was partially reversed by the ferrostatin‐1 (Figure S4Q–T). Then, we evaluated endothelial functionality through a series of assays: cell migration, scratch tests and tube formation assays. The results demonstrated that MEXOs‐shUSP4 significantly impaired cell migration and angiogenesis when compared with the MEXOs‐shNC (Figure S4A–F). Furthermore, the inhibition of USP4 negated the enhanced cellular viability typically conferred by MEXOs‐shNC, as evidenced by Brdu assays (Figure S4G,H).

Additionally, WB analysis substantiated that MEXOs‐shUSP4 was less effective at inhibiting ferroptosis events in HUVECs, evidenced by increased ACSL4 and decreased SLC7A11, GPX4 and FTH1 levels. These changes were further validated through treatments with ferroptosis inducers and inhibitors. Additionally, MEXOs‐shUSP4 treatment significantly reduced the expression of HO1 and SOD1, indicating a diminished resistance to oxidative stress (Figure S4Q,R). DHE staining and DCFH‐DA probe labelling demonstrated that the antioxidative stress and anti‐lipid peroxidation capacities of MEXOs‐shUSP4 were compromised compared with those of MEXOs‐shNC (Figure S4I–L,O,P). The results of the apoptosis flow cytometry assay demonstrated that MEXOs‐shUSP4 diminished the capacity of MEXOs to impede late apoptosis (Figure S4M,N). The levels of GSH, MDA and Fe^2+^ suggesting that MEXOs contribute to the inhibition of ferroptosis in HUVECs by delivering USP4, which enhances cellular resistance against oxidative challenges (Figure S4U). All of those results proved that MEXOs inhibit ferroptosis by shuttling USP4, protecting the function of HUVECs.

### 2.9 USP4 interacts with and stabilises ARNTL

**FIGURE 7:**
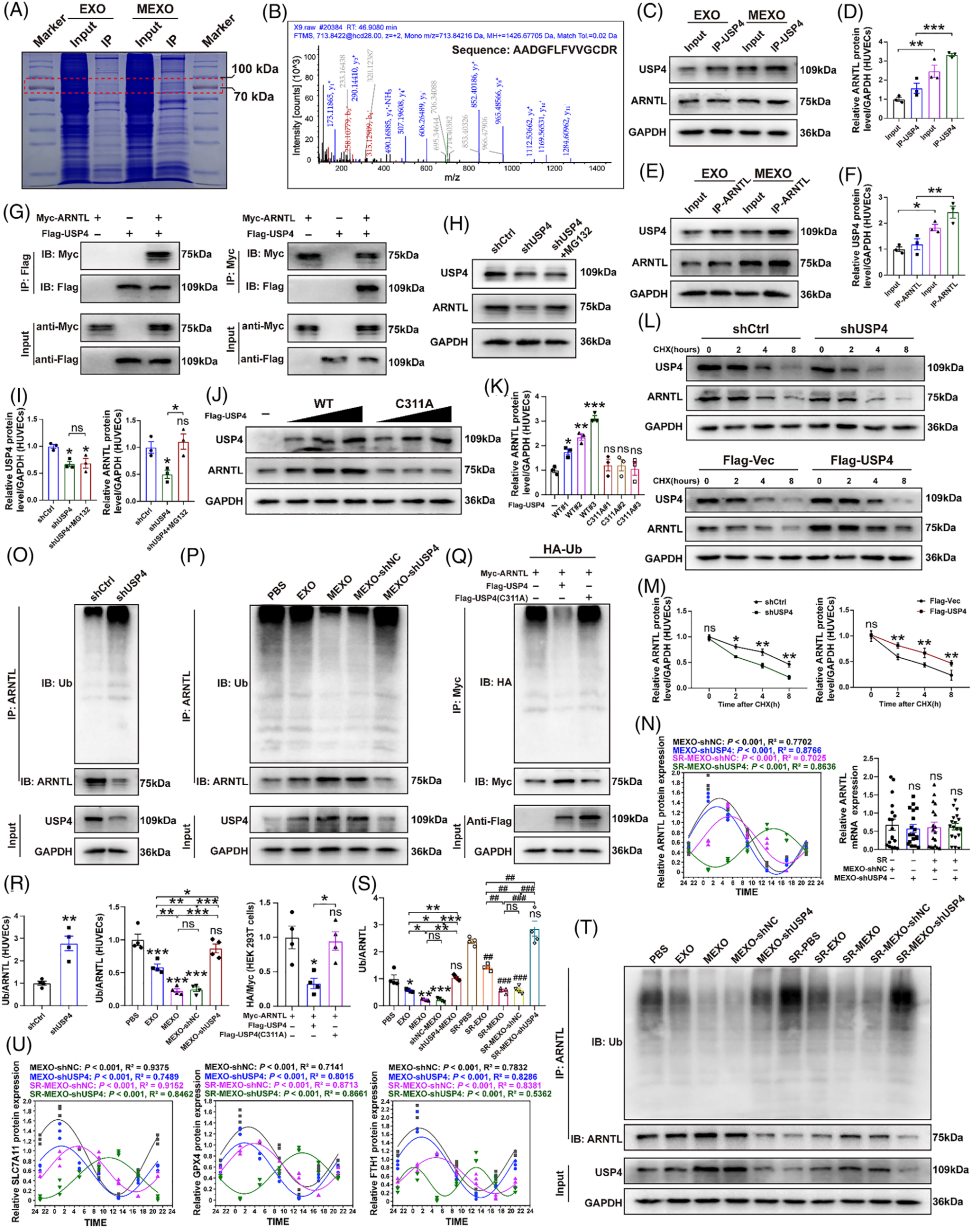
USP4 interacts with and stabilises ARNTL by inhibiting ARNTL ubiquitination. (A) Coomassie blue staining of protein gels after IP (*n* = 3/group). (B) IP/MS analysis indicated that ARNTL is an interacting protein that binds to USP4. (C–F) Endogenous protein interactions were confirmed in HUVECs lysates by immunoprecipitation with anti‐USP4 or ARNTL, followed by immunoblotting with anti‐ARNTL or USP4, respectively (*n* = 3/group). (G) Exogenous protein interactions were confirmed in HEK 293T cells. Lysates from HEK 293T cells transfected with Flag‐tagged USP4 and Myc‐tagged ARNTL plasmids were immunoprecipitated with anti‐Flag or anti‐Myc, respectively, and assessed by immunoblotting with anti‐Myc (ARNTL) and anti‐Flag (USP4) (*n* = 3/group). (H and I) WB analysis of ARNTL and USP4 protein levels in HUVECs transfected with shUSP4 with or without treatment with proteasome inhibitor MG132 (*n* = 3/group). (J and K) HUVECs were transfected with increasing amounts of Flag‐tagged USP4 (WT or C311A mutant), and cell lysates were assessed by immunoblotting with anti‐ARNTL and anti‐Flag (*n* = 3/group). (L and M) ARNTL protein levels in shCtrl and shUSP4 HUVECs were evaluated by immunoblotting in the presence of CHX (10 µg/mL) for indicated timepoint (*n* = 3/group). ARNTL protein levels in Flag‐Vec and Flag‐USP4 HUVECs were evaluated by immunoblotting in the presence of cycloheximide (CHX, 10 µg/mL) for indicated timepoint (*n* = 3/group). (O and R) Lysates from HUVECs transfected with shCtrl or shUSP4 were immunoprecipitated and assessed with indicated antibodies (*n* = 4/group). Quantification of relative levels of ubiquitin‐ARNTL. (P) Lysates from HUVECs were immunoprecipitated with anti‐ARNTL followed by immunoblotting with anti‐Ub and anti‐ARNTL (*n* = 4/group). (Q) Lysates from HEK 293T cells transfected with Flag‐tagged USP4 (WT) or Flag‐tagged USP4 (C311A), together with HA‐tagged Ub and Myc‐tagged ARNTL, were immunoprecipitated with anti‐Myc and immunoblotted with anti‐HA and anti‐Myc (*n* = 4/group). (N) Circadian rhythm patterns for the 24 h cycle of ARNTL using an ELISA assay (*n* = 3 mice/group). The ARNTL mRNA level in flap after MEXO‐shNC and MEXO‐shUSP4 treatment with or without SR (*n* = 18 mice/group). (U) Circadian rhythm patterns for the 24 h cycle of SLC7A11, GPX4 and FTH1 using an ELISA assay (*n* = 3 mice/group). (S and T) Skin flap lysates from mice in indicated groups were immunoprecipitated with anti‐ARNTL followed by immunoblotting with anti‐Ub and anti‐ARNTL (*n* = 4 mice/group). Quantification of relative levels of Ub‐ARNTL. One‐way ANOVA was used for analysis that involved more than two groups; bar graph shows the mean ± SEM; *p < .05, **p < .01 and ***p < .001, ns = not significant.

To elucidate the molecular mechanisms by which USP4 regulates circadian rhythm and ferroptosis, we first employed immunoprecipitation–mass spectrometry (IP–MS) to identify its interacting proteins (Figure 7A,B). This approach revealed ARNTL, a core circadian clock component^34^ and a known repressor of ferroptosis, as a potential binding partner.^35^ Subsequent co‐IP assays in HUVECs confirmed a robust endogenous interaction between USP4 and ARNTL, which was reciprocally validated (Figure 7C–F). This interaction was further corroborated in HEK 293T cells using epitope‐tagged proteins, where Flag‐USP4 co‐precipitated with Myc‐ARNTL and vice versa (Figure 7G).

We next investigated the functional consequence of this interaction. Knockdown of USP4 in HUVECs led to a reduction in ARNTL protein levels, which could be rescued by the proteasome inhibitor MG132, implicating USP4 in regulating the proteasomal degradation of ARNTL (Figure 7H,I). The stabilisation of ARNTL was dependent on the deubiquitinase activity of USP4, as overexpression of wild‐type USP4, but not a catalytically inactive mutant (C311A), increased ARNTL protein abundance (Figure 7J,K). Consistent with this, cycloheximide (CHX) chase assays demonstrated that USP4 overexpression hindered, while its silencing accelerated, ARNTL degradation (Figure 7L,M). Mechanistically, USP4 was found to deubiquitinate ARNTL, as its silencing in HUVECs enhanced ARNTL ubiquitination (Figure 7O). Treatment with MEXOs, which deliver USP4, down‐regulated ARNTL ubiquitination and degradation, an effect that was reversed when MEXOs carrying shUSP4 were applied (Figure 7P). This activity was crucial, as wild‐type USP4, but not the C311A mutant, reduced ARNTL ubiquitination in HEK 293T cells (Figure 7Q).

These in vitro findings were consistent in a mouse model of skin flap after SR. MEXOs‐shUSP4 significantly decreased ARNTL protein levels without affecting its mRNA, confirming post‐translational regulation (Figure 7N). Furthermore, ARNTL was more highly ubiquitinated and degraded in flaps treated with MEXOs‐shUSP4 after SR (Figure 7S,T). Beyond stabilising ARNTL levels, USP4 was critical for maintaining its circadian expression rhythm. Silencing USP4 dampened the rhythm, reducing the mesor and amplitude while delaying the acrophase of ARNTL expression in the flap post‐SR (Figure 7N), which consequently led to circadian fluctuations in ferroptosis‐related proteins (Figure 7U). In conclusion, USP4 interacts with and stabilises ARNTL through deubiquitination, thereby regulating its circadian oscillation and executing clock‐rhythmic control over ferroptosis.

### 2.10 Exosomes that contain USP4 improve skin flap circadian rhythm by stabilising ARNTL

**FIGURE 8:**
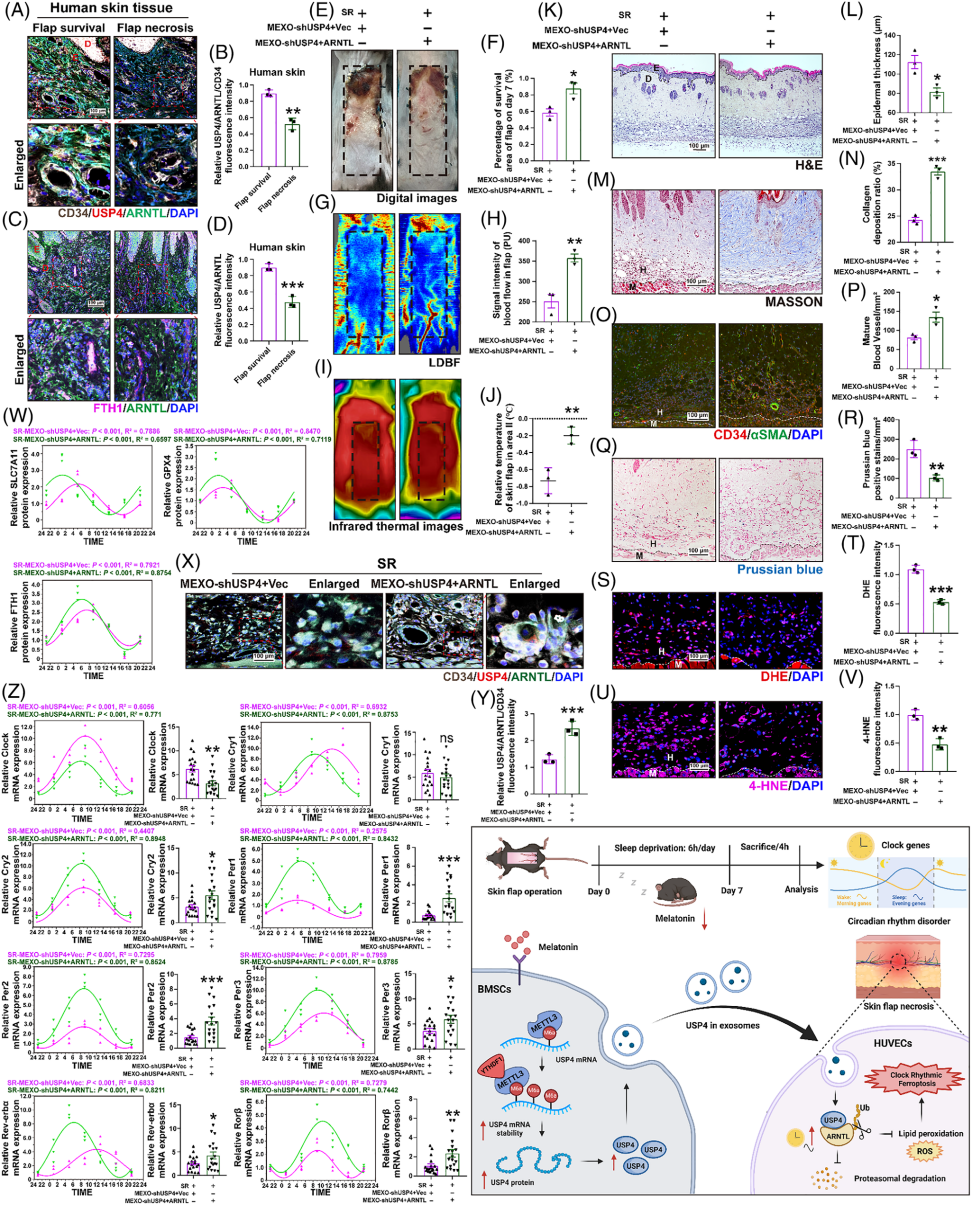
Exosomes containing USP4 improve skin flap circadian rhythm weakened by sleep restriction through stabilising ARNTL. (A and B) The relative expression of USP4 (red)/ARNTL (green) in vascular endothelial cell (CD34, white), and FTH1 (red)/ARNTL (green) in human flap was analysed by IF (*n* = 3 patients/group, scale bar = 100 µm). (E and F) The necrotic area was imaged on day 7 after SR, and the percentage of survival area was analysed (*n* = 3 mice/group). (G and H) Subcutaneous blood flow was detected by LDBF, and the intensity signal of blood flow was analysed (*n* = 3 mice/group). (I and J) Skin temperature was monitored using IRT, the flap temperature was analysed (*n* = 3 mice/group). (K and L) Representative HE images of flap in different groups. The skin and epidermal thickness was determined (*n* = 3 mice/group, scale bar = 100 µm). (M and N) Representative Masson's trichrome images, and the quantification of the collagen deposition (*n* = 3 mice/group). (O and P) Representative IF images for CD34 (red) and α‐SMA (green) of flap. The number of CD34 and α‐SMA‐positive blood vessels (mm^2^) were counted and analysed (*n* = 3 mice/group, scale bar = 100 µm). (Q and R) Prussian blue staining was used to detect the iron ion content (*n* = 3 mice/group, scale bar = 100 µm). (S and T) Frozen sections of the skin tissues were stained with DHE (red, *n* = 3 mice/group, scale bar = 100 µm). (U and V) The expression of 4‐HNE (red) in flap was analysed by IF (*n* = 3 mice/group, scale bar = 100 µm). (W) Circadian rhythm patterns for the 24 h cycle of SLC7A11, GPX4 and FTH1 using an ELISA assay (*n* = 3 mice/group). (X and Y) The relative expression of USP4 (red)/ARNTL (green) in vascular endothelial cell (CD34, white) in flap was analysed by IF (*n* = 3 mice/group, scale bar = 100 µm). (Z) Circadian rhythm patterns for the 24 h cycle of clock genes (*n* = 3 mice/group). Average of clock genes were analysed on the right (*n* = 18 mice/group). Student's *t*‐test was performed to compare two groups; bar graph shows the mean ± SEM; *p < .05, **p < .01 and ***p < .001, ns = not significant.

In human flap tissue, USP4 robustly co‐localised with ARNTL in vascular endothelial cell within viable areas, but this interaction was markedly disrupted in necrotic regions. Accompanying this disruption, the expression of the ferroptosis marker FTH1 was also markedly diminished (Figure 8A–D). These findings suggest that human flap survival involves mutual regulation between ARNTL and USP4, influencing ferroptosis levels. To establish the central role of ARNTL, we performed two key experiments. First, ARNTL knockdown under normal sleep conditions recapitulated the phenotype of clock rhythmic ferroptosis and flap necrosis, thereby demonstrating its sufficiency in driving this process. The shARNTL group exhibited a same necrotic flap area, along with decreased perfusion and skin temperature, relative to the SR group (Figure S5A–F). Consistent with this, associated biomarkers confirmed enhanced ferroptosis and circadian rhythm disruption (Figure S5G–L). Second, we found that overexpressing ARNTL could effectively rescue the flap necrosis phenotype even in mice with USP4 impairment (induced by MEXOs‐shUSP4). ARNTL overexpression significantly improved flap survival compared with MEXOs‐shUSP4 treatment alone, as demonstrated by a reduced necrotic area, enhanced blood flow signals and elevated skin temperature (Figure 8E–J). IF staining further confirmed that ARNTL promoted angiogenesis after MEXOs‐shUSP4 treatment (Figure 8O,P) and increased the co‐localisation of USP4 and ARNTL expression in vascular endothelial cells (Figure 8X,Y). HE and Masson's trichrome staining revealed that ARNTL treatment restored impaired skin barrier function and collagen deposition, accompanied by a marked alleviation of oxidative stress in the flap tissue (Figure 8K–N and 8S–V). Moreover, ARNTL overexpression counteracted ferroptosis induced by MEXOs‐shUSP4, as indicated by decreased iron ion levels (Figure 8Q,R) and the restoration of clock rhythmic ferroptosis (Figure 8W).

Circadian rhythms are self‐sustaining oscillatory phenomena that align with the natural 24‐h cycle.^36^ The transcription factor ARNTL, integral to the mammalian circadian clock, orchestrates clock‐controlled gene expression. To dissect circadian rhythm dynamics in flap, we analysed clock genes including the positive regulator Clock; negative regulators Cry1, Cry2, Per1, Per2 and Per3; and feedback loop genes Rev‐erbα and Rorβ (Figure 8Z). Treatment interventions significantly altered their rhythmic parameters with 24‐h cycle. Notably, Clock exhibited increased mesor and amplitude in the MEXOs‐shUSP4 group, but this effect was mitigated in the MEXOs‐shUSP4 + ARNTL group, reducing circadian oscillation. Conversely, MEXOs‐shUSP4 markedly decreased mesor and amplitude in negative regulators (Cry2, Per1, Per2, Per3) and feedback loop genes (Rev‐erbα, Rorβ), which was rescued by ARNTL overexpression. Acrophase remained unaltered for most genes except Cry1 and Rev‐erbα. Different clock gene types exhibited distinct responses to SR. Critically, MEXOs—which stabilise ARNTL via USP4 delivery—improved the circadian rhythm of downstream clock genes, reduce the clock rhythmic ferroptosis of skin flap.

### 2.11 m6A‐dependent USP4 exosomes inhibit ferroptosis via stabilising ARNTL, protecting the function of HUVECs

Functional rescue experiments confirmed the critical role of the USP4–ARNTL axis in conferring protection. Overexpression of ARNTL in HUVECs effectively reversed the impairments in cell migration, angiogenesis and proliferation induced by MEXOs‐shUSP4 under OGD and H_2_O_2_ stress (Figure S6A–H). Concurrently, ARNTL overexpression mitigated the elevated oxidative stress, lipid peroxidation, apoptosis and ferroptosis levels in HUVECs caused by USP4 deficiency (Figure S6I–R). These results demonstrate that exosomes containing USP4 protect HUVECs function primarily by stabilising ARNTL to inhibit ferroptosis.

To elucidate how USP4 is up‐regulated in exosomes, we investigated the regulatory role of N6‐methyladenosine (m6A) modification.^37^ We found that MT treatment significantly enhanced global m6A levels in BMSCs (Figure S7A, B). Methylated RNA IP (MeRIP)‐qPCR specifically confirmed the enrichment of m6A‐modified USP4 mRNA, which was more pronounced after MT treatment (Figure S7C). Mechanistically, the m6A ‘writer’ METTL3 interacted with USP4 mRNA to facilitate its methylation (Figure S7E). This modification, in turn, recruited the nuclear reader YTHDF1,^38^ which binds to USP4 mRNA and promotes its translation, as evidenced by RNA IP (RIP)‐qPCR (Figure S7D). Consequently, the expression levels of USP4, YTHDF1 and METTL3 were consistently elevated in both MT‐treated BMSCs and the exosomes they secreted (Figure S7F–K). These findings establish that MT treatment enhances the m6A methylation of USP4 mRNA via the METTL3/YTHDF1 axis, thereby boosting USP4 protein expression in BMSCs and their exosomes.

## 3 DISCUSSION

This study establishes that SR induces clock rhythmic ferroptosis, compromising skin barrier integrity and flap survival. We demonstrate that MEXOs outperform conventional exosomes or MT alone in promoting repair. The therapeutic superiority of MEXOs depends on m6A‐mediated USP4 enrichment, which stabilises the circadian regulator ARNTL through deubiquitination, thereby restoring circadian rhythmicity and suppressing ferroptosis. These findings identify MEXOs as a promising therapeutic strategy for flap recovery and elucidate their mechanistic basis.

Random‐pattern skin flaps, while convenient in reconstructive surgery,^39^ face significant survival challenges due to inadequate blood supply^40^ and ischemia–reperfusion injury where oxidative stress plays a critical role.^41^ Although SR is known to compromise skin barrier integrity,^35^ its specific impact on flap necrosis remained unclear until our clinical analysis of 344 patients established SR as an independent risk factor while revealing concomitant activation of ferroptosis and circadian pathways in necrotic flaps. This finding persisted after multivariate adjustment for key confounders, including sedative use, haemodialysis, comorbidities and so on. This highlights that impaired sleep health increases the risk of necrosis independently of other clinical factors. Importantly, the clinical interpretation of SR warrants further nuance. The ‘SR’ identified by the Pittsburgh Sleep Quality Index (PSQI) questionnaire in hospitalised patients likely represents a composite clinical state of impaired sleep health, encompassing not only insufficient sleep duration/quality but also the influences of pain, anxiety, underlying disease severity and the hospital environment itself.^42^ *^,^* ^43^ Therefore, our results should be interpreted as indicating that a pre‐operative state of impaired sleep health is a significant risk factor for flap necrosis. Notably, this clinical condition may share a common pathological pathway with the more controlled SR modelled in mice—namely, the disruption of circadian rhythms and the induction of clock rhythmic ferroptosis.

To address these challenges, we turned to MT—a circadian regulator disrupted by SR^23^ *^,^* ^44^ with demonstrated antioxidant and reparative properties^45^ but limited by poor pharmacokinetics including short half‐life and low oral bioavailability (<15%)^46^ and ineffective postoperative sleep improvement.^9^ Building on previous success with adipose mesenchymal stem cell (MSC)‐derived exosomes to promote flap survival,^47^ we developed MEXOs that overcome these limitations through local delivery. The results demonstrated that MEXO promoted flap survival, particularly in cases of flap necrosis induced by SR. Emerging evidence reveals a complex bidirectional relationship between sleep and ferroptosis, where SR activates ferroptosis pathways that may further disrupt sleep architecture.^48^, ^49^, ^50^, ^51^ Given the shared centrality of oxidative damage in both MT's therapeutic mechanisms and ferroptosis pathogenesis—an understudied ROS‐dependent cell death pathway in skin flaps^52^, ^53^—we investigated this connection. Ferroptosis execution depends critically on lipid peroxidation, while antioxidant systems including GPX4 and system xc− provide multi‐level protection against this process.^54^, ^55^ Our examination of these parameters in flaps after SR demonstrated ferroptosis activation in both clinical patients and animal models. Notably, we discovered these ferroptosis levels exhibit circadian oscillations, leading us to define this phenomenon as ‘clock rhythmic ferroptosis’. In addition to the conventional understanding of ferroptosis, the activation of this process can exhibit significant variation in dynamic terms.

Our findings demonstrate MEXOs’ superior efficacy in suppressing clock rhythmic ferroptosis. Since exosomes are known to deliver functional proteins to recipient cells^56^ *^,^* ^57^—such as ITIH4 for regulating inflammation^58^ and microRNAs for enhancing wound healing^59^—we investigated their protein cargo. We identified USP4 as markedly up‐regulated in MEXOs compared with EXOs, while its expression was significantly reduced in both human and murine flaps following SR. This inverse correlation suggested USP4's pivotal role in regulating clock rhythmic ferroptosis, with MEXOs potentially compensating for SR‐induced USP4 deficiency through targeted delivery. This hypothesis was confirmed when USP4 silencing substantially diminished MEXOs’ protective effects in vivo and in vitro. Although USP4 is established as a deubiquitinase involved in DNA damage response through p53/NF‐κB regulation^60^ *^,^* ^61^ and in cancer progression^62^ via TGF‐β1/MMP‐9 activation, its function in circadian‐related pathologies remains unexplored.^63^ We therefore focused on its deubiquitination activity in regulating clock components in flap, ultimately revealing how USP4 stabilises core clock proteins to establish the circadian pattern of ferroptosis fluctuations.

Mechanistically, we identified ARNTL as a key interacting partner of USP4. While ARNTL is established as a core circadian regulator governing iron metabolism^64^ and cell death pathways,^1^ and its ferroptosis‐suppressing role has been documented in various disease contexts,^65^ *^,^* ^66^ its functional connection with USP4 in SR‐induced ferroptosis remained unexplored.^35^ *^,^* ^67^ *^,^* ^68^ Here, we demonstrate that USP4 binding stabilises ARNTL through deubiquitination, creating an anti‐ferroptosis mechanism with inherent circadian properties. Concurrently, we found that the flap exhibited clock rhythmic ferroptosis by silencing ARNTL consistent with that after SR, demonstrated the centrality of ARNTL in the regulation of clock rhythmic ferroptosis. Additionally, this USP4–ARNTL axis was substantiated by their correlated high expression in viable human flap tissues showing minimal ferroptosis and confirmed through rescue experiments where USP4 mediated clock rhythmic ferroptosis regulation via ARNTL. Molecularly, ARNTL/CLOCK heterodimer drives the core transcriptional–translational feedback loop of circadian genes,^69^ *^,^* ^70^ with our data showing ARNTL overexpression rescues circadian gene expression patterns.^26^ *^,^* ^54^ Thus, ARNTL stability fluctuations emerge as the crucial determinant orchestrating clock rhythmic ferroptosis in flaps.

To elucidate how MT pretreatment up‐regulates USP4 in BMSCs, we investigated m6A—the most prevalent eukaryotic mRNA modification.^37^ *^,^* ^71^ *^,^* ^72^ We identified a METTL3‐mediated mechanism where MT‐induced YTHDF1 enhances USP4 mRNA stability and translation through m6A modification,^38^ *^,^* ^73^ consistent with emerging evidence of m6A‐mediated regulation of deubiquitinating enzymes in various pathological contexts.^13^ *^,^* ^74^, ^75^, ^76^ This results in USP4‐enriched exosomes that mediate therapeutic effects in flap repair. While this study preliminarily establishes USP4‐mediated inhibition of clock rhythmic ferroptosis as a mechanism for flap survival, its full functional scope warrants further investigation using USP4 knockout genetic models, and its clinical translation calls for future safety studies involving repeat‐dose toxicity and long‐term monitoring in large animal models. Our findings highlight MEXOs as an effective USP4 delivery system, though other deubiquitinating enzymes may contribute to this process. Future research should optimise USP4‐specific exosome engineering and explore additional regulatory modifications, potentially enabling dynamic ferroptosis modulation for enhanced therapeutic outcomes in flap repair and related applications.

## 4 CONCLUSIONS

This study demonstrates that SR induces clock rhythmic ferroptosis in skin flaps, leading to impaired barrier function and tissue necrosis. We identify MEXOs as an effective therapeutic strategy that outperforms both basic exosomes and MT alone. The protective effects of MEXOs are mediated through m6A‐dependent enrichment of USP4, which stabilises the core clock protein ARNTL by inhibiting its ubiquitination, thereby restoring circadian rhythms and suppressing ferroptosis. These findings establish MEXOs as a promising cell‐free therapy for enhancing flap survival under conditions of circadian disruption.

## 5 MATERIALS AND METHODS

### 5.1 Patients with skin flap surgery

This retrospective study, which covered a period of 10 years, analysed data from 344 patients who underwent flap surgery. Investigators collected demographic characteristics and clinical baseline data from the hospital electronic case system to partially adjust for potential confounders, which have the capacity to directly impact flap survival. It is important to note that, due to the constraints of the retrospective study design, specific anxiety/depression scales were not routinely available for all cases. Therefore, in our multivariate model, we included ‘sedative use’ as a key clinical proxy for anxiety management. Upon admission, the patient was evaluated by a nurse using a predetermined scale (the PSQI), with the results (normal or insomnia) documented on the admission evaluation form.

The PSQI serves as an effective instrument for assessing sleep quality and patterns. Recent expert consensus on standard insomnia research assessments has endorsed it as a primary metric for global sleep and insomnia symptoms.^7^ *^,^* ^8^ Comprising seven components, the questionnaire evaluates sleep quality and distinguishes ‘poor’ from ‘good’ sleep. These include subjective sleep quality, sleep latency, sleep duration, habitual sleep efficiency, sleep disturbances, use of sleep medication and daytime dysfunction over the past month. The evaluating nurses underwent consistent training in administering this scale, and the outcome was documented as a binary variable within a structured data field. This study was conducted in accordance with the Declaration of Helsinki II and received approval from the Ethics Committee of our affiliated hospital (Protocol KY2025 ‐ R392).

### 5.2 Random‐pattern skin flap model

Male C57BL/6J mice (6–8 weeks old, 20–30 g) were selected to avoid estrogen's impact on flap necrosis.^77^ Animals were obtained from our university's experimental centre and handled per national guidelines (Protocol wydw2019‐0729). They were kept under controlled conditions (21–25°C, 50–60% humidity, 12‐h light/dark cycle) and provided with food and water ad libitum. Under pentobarbital anaesthesia (50 mg/kg, i.p.), a 1.5 × 4.5 cm^2^ caudal‐based flap was surgically created on the dorsum.^78^ After removing visible blood vessels, the flap was repositioned and sutured, followed by iodophor disinfection. This model replicates human ischemic flap pathophysiology.^79^

### 5.3 SR protocol

The SR group underwent a 7‐day intervention post‐surgery: sleep was suppressed via gentle tactile stimulation during 6‐h daily sessions (8:00 AM–2:00 PM), while controls slept freely.^80^ Mice were euthanised on day 7, and flap tissues (divided into proximal I, intermediate II and distal III regions) and right atrial blood were collected at 4‐h intervals across six time points. Flap survival area, blood perfusion and skin temperature were assessed on day 7 (2:00–5:00 PM). A 1 × 1 cm^2^ sample from region II was excised, rinsed with PBS and processed as required.

### 5.4 Analysis of skin flap survival area

Macroscopic properties of the flap, including appearance, colour and hair condition, were meticulously observed and documented on day 7 post‐operation. On the same day, the percentage of flap survival area was quantitatively assessed utilising Image J software (National Institutes of Health, Bethesda, MD). To determine the percentage of flap survival, the following equation was employed: % survival = survival area ÷ total area × 100%.

### 5.5 Laser Doppler blood flow

The blood supply and vascular network integrity of the flap were visualised using laser Doppler blood flow (LDBF). On day 7 post‐surgery, a laser Doppler instrument from Moor Instruments (Axminster, UK) was employed to assess the blood supply and map the small vessel networks within the flap. Blood flow was quantitatively evaluated in terms of perfusion units (PU), utilising the moor LDI Review software (version 6.1; Moor Instruments). The blood flow measurements were conducted three times for each mouse and used for subsequent statistical analysis.

### 5.6 Infrared Thermography

The flap temperature was monitored using IRT. On day 7 after the operation, the flap temperature was photographed using a smartphone‐based camera (FLIR, USA) according to the previous study protocol. The relative temperatures in area II were quantified using FLIR supporting software. Each mouse was measured three times, and the average was used for statistics.

### 5.7 Histological analysis

Histopathologic analysis was conducted on tissue samples harvested from area II of the flap s on day 7 post‐operation. The specimens were fixed in 4% paraformaldehyde for a full 24 h. The embedded tissues were then sectioned and subjected to staining processes critical for detailed histological examination: HE staining, which highlights cellular and nuclear features, and Masson's trichrome staining, which differentiates collagen fibres within the connective tissue, thereby allowing assessment of the epidermal thickness and the integrity of collagen structures. Additionally, the presence of iron deposits within the tissue was evaluated using the Prussian Blue Iron Stain Kit (YEASEN), following the manufacturer's protocols. The stained sections were meticulously examined under a Nikon microscope.

### 5.8 Immunohistochemistry

Histological sections of skin tissues from area II of the flap in each experimental group underwent a series of preparative steps. Initially, sections were deparaffinised in xylene and progressively rehydrated through a series of graded ethanol solutions. After rehydration, we performed antigen retrieval by heating the sections in 10.2 mM sodium citrate buffer at 95°C for 10 min. Subsequent steps included washing the sections, quenching endogenous peroxidase activity with 3% H_2_O_2_, and blocking with 10% bovine serum albumin (BSA) in PBS for 60 min to prevent nonspecific antibody binding. The sections were then incubated overnight at 4°C with a primary antibody targeting SLC7A11 (Abcam, ab307601). Then, sections were exposed to an HRP‐labelled secondary antibody. The presence of the target antigen was then visualised using a DAB detection Kit, producing a brown precipitate at the site of the antigen‐antibody reaction. The stained samples were examined under a Nikon microscope to assess the localisation and expression levels of SLC7A11.

### 5.9 IF staining

Flap tissue sections were deparaffinised and rehydrated as previously described. Antigen retrieval was performed with 10.2 mM sodium citrate buffer at 95°C for 10 min. After cooling to room temperature, the sections were blocked for 60 min using 10% BSA. They were then incubated overnight at 4°C with the following primary antibodies: ACSL4 (Affinity; DF12141), FTH1 (Proteintech; 10727‐1‐AP), CD34 (Immunoway; YM8525), α‐SMA (Immunoway; YM0011), 4‐HNE (Abcam; ab275359), USP4 (Proteintech; 66822‐1‐Ig) and anti‐FLAG (Proteintech; 66008‐4‐Ig). Subsequently, a secondary antibody was applied for 1 h at 37°C, followed by nuclear staining with DAPI (Abcam). Imaging of positive cells was conducted using a Nikon fluorescence microscope.

### 5.10 DHE staining

Excised skin tissues were processed in a 30% sucrose solution until sinking, which served as the criterion for sufficient penetration. Subsequently, the tissues were embedded in OCT compound. The embedded tissues were then sectioned into 20 µm slices using a cryostat and allowed to thaw. To reduce autofluorescence, sections were incubated with a quenching reagent for 5 min. ROS detection involved staining the sections with DHE (Beyotime) at 37°C for 30 min. Subsequently, sections were stained with DAPI (Abcam) to visualise nuclei. The final examination of the sections was performed using a Nikon fluorescence microscope, capturing images of positively stained cells.

### 5.11 Cell culture

MSCs were isolated from mouse bone marrow using established protocols.^13^ Under sterile conditions, the femurs and tibiae from the lower limbs were dissected, and attached muscle along with adipose tissues were carefully removed. The marrow cavity was repeatedly flushed with culture medium via a syringe to harvest the bone marrow. The collected rinsing fluid was passed through a 70 µm sterile filter (Corning) to eliminate debris and then subjected to centrifugation at 200 g for 5 min to pellet the cells. The resulting cell pellet was resuspended and seeded at 1 × 10^6^ cells/mL into a 25 cm^2^ culture flask. Cells were maintained in DMEM medium (Gibco) containing 10% foetal bovine serum (FBS; Gibco) and 1% penicillin‐streptomycin (Gibco) at 37°C with 5% CO_2_. After 24 h, non‐adherent cells were removed, and the adherent population was continuously cultured until reaching 80–90% confluence, with the medium refreshed every other day. BMSCs at passages 3–5 were used for subsequent experiments.

Then, BMSCs were pretreated with MT (1 µmol/L) in exosome‐depleted 10% FBS‐ (System Biosciences) containing culture medium (Gibco) for 48 h. The surface biomarkers of BMSCs were identified using flow cytometry with FITC‐and PE‐conjugated antibodies. CD90 and CD44 were confirmed as positive markers, while CD45 and CD34 were negative markers (BD Biosciences), consistent with the manufacturer's instructions.

HUVECs were cultured in an incubator using DMEM supplemented with 10% FBS and 1% penicillin/streptomycin solution. The HUVECs underwent OGD by being cultured in a sugar‐free medium. After being placed in a sealed chamber and flushed with the gas mixture (95% N_2_/5% CO_2_) for 15 min, the cells were maintained in the sealed chamber within a 37°C incubator for 4 h. Subsequently, the medium was changed to normal medium, and the cells were cultured in a standard incubator for another 6 h.^78^ Finally, H_2_O_2_ (300 µmol/L) was added to the HUVECs to induce an oxidative stress environment for 24 h.

### 5.12 Exosomes isolation and identification

Exosomes were isolated and purified according to the classical differential centrifugation/ultracentrifugation protocol, guided by the MISEV2018 guideline. When BMSCs reached 80% confluency, the medium was replaced with exosome‐depleted 10% FBS, with or without MT, for 48 h. The conditioned medium was then collected and processed through sequential centrifugations: first at 300×*g* for 10 min, then at 2000×*g* for 30 min at 4°C, to eliminate dead cells, apoptotic bodies and debris. The resulting supernatant was further cleared by centrifugation at 10 000×*g* for 30 min and filtered through a  .22‐µm Steritop™ filter (Millipore). The filtered solution was ultracentrifuged at 100 000×*g* for 70 min at 4°C using a CP100NX ultracentrifuge (Eppendorf Himac Technologies) to obtain the exosome pellet. After supernatant removal, the pellet was washed with sterile PBS and subjected to a second round of ultracentrifugation under the same conditions. Finally, the purified exosomes were resuspended, aliquoted and either used directly in subsequent assays or stored at −80°C.

Exosome size distribution was analysed using a NanoSight NS300 instrument (Malvern Panalytical Ltd.), which determines particle size via nanoparticle tracking analysis of Brownian motion in suspension. Morphological assessment was performed using a Tecnai 12 transmission electron microscope (Philips).

WB analysis was conducted to identify exosomal markers, specifically CD81 (Abcam; ab219209), CD9 (Abcam; ab236630) and TSG101 (Abcam; ab125011). Additionally, calnexin (Proteintech; 10427‐2‐AP), a negative marker for exosomes, was also assessed to evaluate the purity of the exosome preparations.

Exosomes suspended in PBS were incubated with Dil solution (Beyotime) to facilitate the detection of exosome uptake. The Dil‐labelled exosomes were then co‐cultured with HUVECs for 24 h. After incubation, HUVECs were washed with PBS to remove any unbound exosomes and fixed with 4% paraformaldehyde. The internalisation of Dil‐labelled exosomes was then imaged using a Nikon fluorescence microscope.

### 5.13 Cell transfection

Constructs expressing Flag‐tagged USP4, Flag‐tagged USP4 C311A, Myc‐tagged ARNTL and HA‐tagged ubiquitin were generated by cloning the respective open reading frames into expression vectors with an N‐terminal Flag or Myc tag. Lipofectamine 3000 reagent (Invitrogen) was used according to the manufacturer's protocol to improve transient transfection. To achieve stable knockdown of USP4, lentiviral vectors encoding shRNA targeting USP4 were constructed by GENE, with scrambled shRNA lentiviral constructs serving as negative controls.

### 5.14 Adeno‐associated virus vector packaging

Recombinant adeno‐associated virus 2 (AAV2) vectors were engineered for both ARNTL knockdown and overexpression. For knockdown, ARNTL‐specific shRNA sequences were designed, synthesised and cloned into an AAV2 vector downstream of a U6 promoter. A separate CMV promoter driving ZsGreen expression was included in the same vector for transduction tracking. For overexpression, the ARNTL coding sequence was inserted into an AAV2 vector under the control of a CMV promoter. All constructs were verified by sequencing prior to viral packaging.

### 5.15 Quantitative PCR

Total RNA from skin tissues was extracted with TRIzol reagent (Invitrogen) as instructed. This RNA was used for complementary DNA (cDNA) synthesis with HiScript III RT SuperMix (Vazyme) on a VeritiPro™ Thermal Cycler (Thermo Fisher Scientific, USA). Quantitative PCR (qPCR) then employed Taq Pro Universal SYBR qPCR Master Mix (Vazyme) on a C1000 Touch Thermal Cycler (BIO‐RAD, USA). The relative gene expression levels were calculated using the 2^–ΔΔCt^ method, with ACTB serving as an internal control gene to normalise the expression levels of the target genes.

### 5.16 Immunoprecipitation

HUVECs, HEK 293T cells and skin tissues from flap area II were lysed using RIPA buffer containing protease inhibitors (Beyotime). Prior to IP, antibodies were diluted as specified and pre‐incubated with protein A/G agarose beads (Elabscience) for 2 h at 4°C. The lysates were then subjected to overnight IP at 4°C. The next day, protein A/G beads were added for an additional 2‐h incubation to capture immunocomplexes. After five washes with RIPA buffer to remove non‐specific proteins, the bead‐bound complexes were eluted by boiling and analysed by SDS‐PAGE and WB.

### 5.17 IP coupled with MS

In the IP/MS analysis, bound proteins were eluted by boiling and resolved by SDS‐PAGE. After electrophoresis, proteins were visualised by staining the gels with Coomassie Brilliant Blue (Solarbio). This staining provided clear visualisation and precise excision of the target protein bands. Subsequently, the excised gel slices were analysed by MS.

### 5.18 Measurement of m6A modification

The assessment of m6A modifications in total RNA was performed using an EpiQuik m6A RNA Methylation Quantification Kit (Epigentek) following the manufacturer's instructions. The procedure begins by binding 200 ng of total RNA to the strip wells coated with a high‐affinity RNA binding solution. The m6A residues in the RNA are then specifically recognised and captured by an m6A‐specific antibody, followed by the addition of a detection antibody to facilitate the visualisation of the bound complex. This is achieved colorimetrically by reading the absorbance at a wavelength of 450 nm using a microplate spectrophotometer.

### 5.19 RNA dot blot

Following RNA isolation with RNAiso Plus (TaKaRa) for dot blot analysis, samples were spotted onto nylon membranes (Millipore, INYC00010) and crosslinked by UV irradiation. To assess RNA quality, membranes were stained with methylene blue and destained with ethanol. Prior to immunodetection, a 2‐h block was performed using 5% milk in TBST. The membranes were probed overnight at 4°C with an anti‐m6A primary antibody (Proteintech; 68055‐1‐Ig). After incubation with a secondary antibody for 2 h at room temperature, m6A signals were visualised using the Omni‐ECL™ Femto Light Chemiluminescence Kit (EpiZyme).

### 5.20 RNA IP

To analyse protein‐RNA interactions, RIP was performed using a commercial kit (BersinBio) per the manufacturer's protocol. Briefly, 10^7^ BMSCs were harvested, PBS‐washed and lysed in IP buffer supplemented with protease and RNase inhibitors. After centrifugation, 10% of the lysate was aliquoted as input and stored at −80°C. The remainder was incubated overnight at 4°C with antibodies against YTHDF1 (Proteintech; 17479‐1‐AP) or METTL3 (Proteintech; 15073‐1‐AP), using normal rabbit IgG as a negative control. Subsequently, magnetic protein A/G beads were added to capture the complexes for 1 h at 4°C. Following incubation, the beads were treated with Proteinase K at 55°C for 30 min, and RNA was extracted using RNAiso Plus. RNA enrichment was assessed by quantitative PCR and normalised to the input.

### 5.21 MeRIP‐qPCR

Following isolation with RNAiso Plus, total RNA was subjected to m6A analysis using the EpiQuik™ CUT&RUN m6A RNA Enrichment Kit (Epigentek). Antibody‐bead complexes were prepared by incubating the anti‐m6A antibody (Proteintech; 68055‐1‐Ig) or control IgG with Magna ChIP Protein A/G Magnetic Beads at 4°C for 90 min. From 200 µg of total RNA per IP, 20 µg was reserved as an input control at −80°C. The remainder was incubated overnight at 4°C with the complexes. Subsequent elution was performed twice with a buffer containing 20 mM m6A monophosphate sodium salt. The input and eluted RNA were then purified again using RNAiso Plus, and enrichment was assessed via qPCR following the established protocol.

### 5.22 Determination of ROS

Cellular ROS levels in HUVECs were determined using the oxidation‐sensitive fluorescent probe DCFH‐DA (Beyotime) as per the manufacturer's protocol. The cells were subsequently stained with Hoechst 33342 (Beyotime). Finally, positive signals were captured using a Nikon fluorescence microscope or flow cytometry.

### 5.23 Tube formation assay

The angiogenic activity of HUVECs was evaluated using Confocal Dishes (Corning) precoated with 100 µL/well of Matrix‐Gel™ Basement Membrane Matrix (Beyotime). Initially, HUVECs were stained with the cell‐permeable dye calcein AM for 30 min and then replated onto the precoated Confocal Dishes. The cells were incubated for 4 h at 37°C in a cell culture incubator to allow for capillary‐like tube formation. This formation was subsequently captured under a Nikon fluorescence microscope.

### 5.24 Cell migration

To assess the in vitro migration activity of HUVECs, cell migration assays were conducted using Transwell inserts with an 8.0 µm polycarbonate membrane (Corning). To assess migration under various treatments, HUVECs were seeded into the upper chambers of a Transwell system and incubated for 24 h at 37°C. Following fixation with 4% paraformaldehyde and staining with crystal violet (Beyotime), the cells that had migrated through the membrane were imaged using a Nikon fluorescence microscope for quantitative and qualitative assessment.

### 5.25 Scratching assay

HUVECs were cultured in a six‐well plate until they reached 90% confluence. To create artificial wounds, a sterile 10 µL pipette tip was used to scrape the cell monolayer. Subsequently, the scraped cells were washed with PBS to remove cellular debris. Cell migration into the wound area was monitored using a Nikon fluorescence microscope and images were captured at 0 and 24 h post‐scraping. The migration distance, indicating the gap between the two edges of the wound, was quantitatively measured using ImageJ software.

### 5.26 Edu staining

The proliferative capacity of HUVECs was assessed using the BeyoClick™ EdU Cell Proliferation Kit (Beyotime) according to the manufacturer's instructions. Cells were incubated with the EdU reagent and then stained with Hoechst 33342 (Beyotime). EdU‐positive cells were finally imaged under a Nikon fluorescence microscope.

### 5.27 Determination of lipid peroxidation, GSH and iron content

The concentrations of lipid peroxidation products, specifically MDA, were quantified in flap and cell lysates using lipid peroxidation detection kits (Solarbio). Additionally, the relative concentration of GSH in the same samples was measured using a GSH assay kit (Solarbio). The iron concentration in flap and cell lysates was determined using an iron assay kit (Abcam).

### 5.28 Enzyme‐linked immunosorbent assay

Blood from the right ventricle of mice was collected from each group starting at 9:00 AM, at 4‐h intervals following the last treatment, over a 24‐h period. The plasma samples were either immediately analysed or stored at −80°C for future analysis. MT and corticosterone levels in the plasma were quantified using mouse‐specific enzyme‐linked immunosorbent assay (ELISA) kits (Elabscience). In addition, the mice flap tissue for MT, Corticosterone, ARNTL (Coibo), SLC7A11 (Coibo), GPX4 (Cusabio) and FTH1 (Cusabio) assays using mouse‐specific ELISA kits according to the time scheme described above.

### 5.29 Western blot analysis

Following dissection from flap area II, skin tissue samples were homogenised in ice‐cold RIPA lysis buffer (Beyotime) containing protease inhibitor cocktail (Beyotime) and phosphatase inhibitor cocktail III (Beyotime). After centrifugation of the homogenates at 20 000×*g* for 30 min at 4°C, the protein concentration in the supernatant was measured with a BCA protein assay kit (Thermo Fisher Scientific). For analysis, 20 µg of total protein per sample was electrophoresed on 10% SDS‐polyacrylamide gels and then electroblotted onto PVDF membranes (Millipore). Following the TBST wash, a 2‐h block was performed using 5% milk in TBST. The membranes were incubated overnight at 4°C with primary antibodies against SLC7A11 (Abcam; ab307601), ACSL4 (Abcam; ab155282), GPX4 (Abcam; ab125066), FTH1 (Zenbio; R23306), HO1 (Abcam; ab68477), SOD1 (Abcam; ab308181), VEGFA (Abcam; ab214424), USP4 (Santa Cruz Biotechnology; sc‐376000), ARNTL (Proteintech; 14268‐1‐AP), anti‐Flag (Abcam; ab205606), anti‐Myc (Abcam; ab206486), Ub (Cell Signaling; 3936S) and GAPDH (Proteintech; 10494‐1‐AP). Subsequently, the membranes were incubated with HRP‐labelled secondary antibodies at room temperature for 2 h. Following detection with an Omni‐ECL™ Femto Light Chemiluminescence Kit (EpiZyme), images were acquired using a fluorescence imaging analysis system. The acquired images were then processed with Image J software (NIH) to determine protein expression levels.

### 5.30 Statistical analysis

All statistical analyses were performed using SPSS 25.0 and GraphPad Prism version 8.0, with results presented as means ± SEM. Normality of data distribution was assessed using the Shapiro–Wilk test, and homogeneity of variances was verified using Levene's test. For comparisons between two groups, if data met assumptions of normality and homogeneity, Student's *t*‐test was applied; otherwise, the non‐parametric Mann–Whitney *U* test was used. For comparisons among more than two groups, one‐way or two‐way analysis of variance (ANOVA) was employed when data were normally distributed with homogeneous variances, followed by Bonferroni's post hoc test for multiple comparisons. If data did not meet parametric assumptions, the Kruskal–Wallis test with Dunn's post hoc analysis was applied. The number of samples for each analysis is detailed in the figure legends. A *p* value < .05 was considered statistically significant.

## AUTHOR CONTRIBUTIONS

Xiaoqiong Jiang, Kailiang Zhou and Jian Xiao conceived and supervised the work. Xiaoqiong Jiang, Yu Wang, Xiangwei Ling, Liangyu Fang, Anqi Ye and Xuanlong Zhang performed in vitro and in vivo experiments. Xiangwei Ling and Yu Wang performed the sleep data analysis. Xiangwei Ling, Kailiang Zhou and Jian Xiao guided the animal experiments. Huiming Deng, Xiaoqiong Jiang, Hao Chen, Xiangwei Ling and Jiangnan Yao assisted in the collection of clinical patient data. Xiaoqiong Jiang, Yu Wang, Xuanlong Zhang and Chaire Tafadzwa performed data analysis and drafted the manuscript.

## FUNDING INFORMATION

This work was supported by the Basic Public Welfare Research Project of Zhejiang Province (Protocol No. LGF22H110002 and LZ23H060001) and the Project of Wenzhou Science and Technology Bureau (Protocol No. Y2023399 and Y20240823).

## CONFLICT OF INTEREST STATEMENT

The authors declare no conflicts of interest.

## ETHICS STATEMENT

This study was conducted following the Declaration of Helsinki and national guidelines for animal care, with approvals obtained from the Ethics Committee of The First Affiliated Hospital of Wenzhou Medical University (KY2025‐ R392) and the Animal Ethics Committee of Wenzhou Medical University (wydw2019‐0729).

## Supporting information



Supporting Information.

## Data Availability

All data supporting the conclusions of this work are provided in the text and figures. Please contact the author for data requests.
