# Supplementary material for: Melatonin‐engineered MSCs‐exosomes deliver USP4 to stabilise ARNTL and inhibit clock rhythmic ferroptosis for enhanced flap survival
Source: Clin Transl Med. 2026 Jan 2;16(1):e70565. doi: 10.1002/ctm2.70565 (PMC12759045; doi:10.1002/ctm2.70565)
Supplement: Supplementary file 1 — Supporting Information. [file CTM2-16-e70565-s001.docx]

**Supplementary Table 1. Rhythm parameters determined by Circa Compar analysis.**

| **Variables** | **Rhythmic-*p*** | **Mesor** | **Amplitude** | **Acrophase** |
| --- | --- | --- | --- | --- |
| **Control** | ＜.001 | 3.87116 ± 0.40472 | 5.81833 ± 0.59208 | 0.943235 |
| **SR** | ＜.01 | 1.21293 ± 0.25011 | 1.87841 ± 0.36703 | 5.566595 |

**Figure 3: Melatonin (skin flaps)**

**Figure 3: Melatonin (plasma)**

| **Variables** | **Rhythmic*-p*** | **Mesor** | **Amplitude** | **Acrophase** |
| --- | --- | --- | --- | --- |
| **Control** | ＜.001 | 63.47655 ±1.65424 | 36.11513 ±2.28907 | 0.5193 |
| **SR** | ＜.01 | 46.52167 ±0.97976 | 12.30464 ± 0.8099 | 9.06804 |

**Figure 7: ARNTL (skin flaps)**

| **Variables** | **Rhythmic-*p*** | **Mesor** | **Amplitude** | **Acrophase** |
| --- | --- | --- | --- | --- |
| **MEXO-shNC** | < .001 | 0.74659 ± 0.059 | 0.73709 ± 0.089 | 3.292 |
| **MEXO-shUSP4** | < .001 | 0.63670 ± 0.038 | 0.67924 ± 0.057 | 2.786 |
| **SR-MEXO-shNC** | ＜.001 | 0.64829 ± 0.047 | 0.47155 ± 0.067 | 5.387 |
| **SR-MEXO-shUPS4** | < .001 | 0.48791 ± 0.023 | 0.38609 ± 0.034 | 14.365 |

**Figure 7: SLC7A11 (skin flaps)**

| **Variables** | **Rhythmic-*p*** | **Mesor** | **Amplitude** | **Acrophase** |
| --- | --- | --- | --- | --- |
| **MEXO-shNC** | < .001 | 0.87270 ± 0.031 | 0.75134 ± 0.044 | 0.699 |
| **MEXO-shUSP4** | < .001 | 0.58791 ± 0.044 | 0.50675 ± 0.065 | 1.351 |
| **SR-MEXO-shNC** | ＜.001 | 0.63170 ± 0.021 | 0.45683 ± 0.031 | 4.619 |
| **SR-MEXO-shUPS4** | < .001 | 0.45367 ± 0.041 | 0.40734 ± 0.039 | 10.977 |

**Figure 7: GPX4 (skin flaps)**

| **Variables** | **Rhythmic-*p*** | **Mesor** | **Amplitude** | **Acrophase** |
| --- | --- | --- | --- | --- |
| **MEXO-shNC** | < .001 | 0.78482 ± 0.050 | 0.54146 ± 0.075 | 2.686 |
| **MEXO-shUSP4** | < .001 | 0.55408 ± 0.036 | 0.48503 ± 0.053 | 1.879 |
| **SR-MEXO-shNC** | ＜.001 | 0.60150 ± 0.025 | 0.42512 ± 0.037 | 3.920 |
| **SR-MEXO-shUPS4** | < .001 | 0.48549 ± 0.022 | 0.37119 ± 0.032 | 13.707 |

**Figure 7: FTH1 (skin flaps)**

| **Variables** | **Rhythmic-*p*** | **Mesor** | **Amplitude** | **Acrophase** |
| --- | --- | --- | --- | --- |
| **MEXO-shNC** | < .001 | 1.00228 ± 0.059 | 0.74749 ± 0.087 | 1.450 |
| **MEXO-shUSP4** | < .001 | 0.81282 ± 0.049 | 0.72495 ± 0.073 | 1.824 |
| **SR-MEXO-shNC** | ＜.001 | 0.61765 ± 0.029 | 0.42953 ± 0.042 | 4.767 |
| **SR-MEXO-shUPS4** | < .001 | 0.46938 ± 0.045 | 0.34134 ± 0.067 | 0.262 |

**Figure 8: SLC7A11 (skin flaps)**

| **Variables** | **Rhythmic-*p*** | **Mesor** | **Amplitude** | **Acrophase** |
| --- | --- | --- | --- | --- |
| **SR-MEXO-shUPS4-Vec** | ＜.001 | 1.24979 ± 0.082 | 0.92766 ± 0.117 | 5.165 |
| **SR-MEXO-shUPS4-ARNTL** | ＜.001 | 1.5208 ± 0.146 | 1.19555 ± 0.211 | 1.133 |

**Figure 8: GPX4 (skin flaps)**

| **Variables** | **Rhythmic-*p*** | **Mesor** | **Amplitude** | **Acrophase** |
| --- | --- | --- | --- | --- |
| **SR-MEXO-shUPS4-Vec** | ＜.001 | 0.86061 ± 0.051 | 0.73563 ± 0.076 | 2.851 |
| **SR-MEXO-shUPS4-ARNTL** | ＜.001 | 1.06934 ± 0.114 | 1.07794 ± 0.168 | 1.677 |

**Figure 8: FTH1 (skin flaps)**

| **Variables** | **Rhythmic-*p*** | **Mesor** | **Amplitude** | **Acrophase** |
| --- | --- | --- | --- | --- |
| **SR-MEXO-shUPS4-Vec** | ＜.001 | 1.66547 ± 0.095 | 0.96766 ± 0.122 | 7.305 |
| **SR-MEXO-shUPS4-ARNTL** | ＜.001 | 1.84944 ± 0.094 | 1.36354 ± 0.126 | 6.134 |

**Figure 8: Clock (skin flaps)**

| **Variables** | **Rhythmic-*p*** | **Mesor** | **Amplitude** | **Acrophase** |
| --- | --- | --- | --- | --- |
| **SR-MEXO-shUPS4-Vec** | ＜.001 | 3.44135 ± 0.263 | 2.7847 ± 0.339 | 7.834 |
| **SR-MEXO-shUPS4-ARNTL** | ＜.001 | 5.78373 ± 0.665 | 4.61928 ± 0.544 | 9.000 |

**Figure 8: Cry1 (skin flaps)**

| **Variables** | **Rhythmic-*p*** | **Mesor** | **Amplitude** | **Acrophase** |
| --- | --- | --- | --- | --- |
| **SR-MEXO-shUPS4-Vec** | ＜.001 | 4.97649 ± 0.336 | 3.91549 ± 0.328 | 9.829 |
| **SR-MEXO-shUPS4-ARNTL** | ＜.001 | 5.71166 ± 0.415 | 4.14541 ± 0.610 | 14.195 |

**Figure 8: Cry2 (skin flaps)**

| **Variables** | **Rhythmic-*p*** | **Mesor** | **Amplitude** | **Acrophase** |
| --- | --- | --- | --- | --- |
| **SR-MEXO-shUPS4-Vec** | ＜.001 | 1.24979 ± 0.082 | 0.92766 ± 0.117 | 5.165 |
| **SR-MEXO-shUPS4-ARNTL** | ＜.001 | 1.5208 ± 0.146 | 1.19555 ± 0.211 | 1.133 |

**Figure 8: Per1 (skin flaps)**

| **Variables** | **Rhythmic-*p*** | **Mesor** | **Amplitude** | **Acrophase** |
| --- | --- | --- | --- | --- |
| **SR-MEXO-shUPS4-Vec** | ＜.001 | 2.62312 ± 0.165 | 2.31706 ± 0.223 | 5.902 |
| **SR-MEXO-shUPS4-ARNTL** | ＜.001 | 0.6697 ± 0.089 | 0.79557 ± 0.123 | 5.000 |

**Figure 8: Per2 (skin flaps)**

| **Variables** | **Rhythmic-*p*** | **Mesor** | **Amplitude** | **Acrophase** |
| --- | --- | --- | --- | --- |
| **SR-MEXO-shUPS4-Vec** | ＜.001 | 3.86152 ± 0.229 | 2.85032 ± 0.267 | 8.780 |
| **SR-MEXO-shUPS4-ARNTL** | ＜.001 | 1.61237 ± 0.119 | 1.12038 ± 0.154 | 8.704 |

**Figure 8: Per3 (skin flaps)**

| **Variables** | **Rhythmic-*p*** | **Mesor** | **Amplitude** | **Acrophase** |
| --- | --- | --- | --- | --- |
| **SR-MEXO-shUPS4-Vec** | ＜.001 | 5.47192 ± 0.568 | 4.55419 ± 0.431 | 9.383 |
| **SR-MEXO-shUPS4-ARNTL** | ＜.001 | 3.24748 ± 0.353 | 2.80049 ± 0.323 | 10.888 |

**Figure 8: Rev-erbα (skin flaps)**

| **Variables** | **Rhythmic-*p*** | **Mesor** | **Amplitude** | **Acrophase** |
| --- | --- | --- | --- | --- |
| **SR-MEXO-shUPS4-Vec** | ＜.001 | 4.25018 ± 0.311 | 3.94587 ± 0.409 | 6.224 |
| **SR-MEXO-shUPS4-ARNTL** | ＜.001 | 2.42719 ± 0.230 | 1.90604 ± 0.282 | 12.011 |

**Figure 8: Rorβ (skin flaps)**

| **Variables** | **Rhythmic-*p*** | **Mesor** | **Amplitude** | **Acrophase** |
| --- | --- | --- | --- | --- |
| **SR-MEXO-shUPS4-Vec** | ＜.001 | 2.55677 ± 0.194 | 1.95021 ± 0.253 | 10.445 |
| **SR-MEXO-shUPS4-ARNTL** | ＜.001 | 1.2411 ± 0.096 | 1.04729 ± 0.140 | 9.121 |

**Supplementary Figure 2: Corticosterone (skin flaps)**

| **Variables** | **Rhythmic-*p*** | **Mesor** | **Amplitude** | **Acrophase** |
| --- | --- | --- | --- | --- |
| **Control** | < .001 | 2.33122 ± 0.293 | 3.32767 ± 0.443 | 15.256 |
| **SR** | < .001 | 9.50138 ± 0.368 | 4.48766 ± 0.487 | 6.666 |
| **SR-MT** | < .001 | 3.95377 ± 0.364 | 4.45855 ± 0.537 | 13.535 |
| **SR-EXO** | ＜.001 | 8.36647 ± 0.411 | 5.72484 ± 0.337 | 9.499 |
| **SR-MEXO** | < .001 | 8.64222 ± 0.366 | 5.11394 ± 0.539 | 13.605 |

**Supplementary Figure 2: Corticosterone (plasma)**

| **Variables** | **Rhythmic-*p*** | **Mesor** | **Amplitude** | **Acrophase** |
| --- | --- | --- | --- | --- |
| **Control** | < .001 | 47.43044 ± 0.60718 | 11.83986 ± 0.87585 | 13.089 |
| **SR** | < .001 | 77.51852 ± 1.75789 | 34.4245 ± 2.54021 | 4.590 |
| **SR-MT** | < .001 | 66.15775 ± 1.51956 | 20.45565 ± 2.26024 | 13.983 |
| **SR-EXO** | ＜.001 | 73.12896 ± 2.52865 | 31.45106 ± 1.9386 | 9.406 |
| **SR-MEXO** | < .001 | 78.151 ± 1.34094 | 28.44238 ± 2.01712 | 14.810 |

**Supplementary Figure 2: SLC7A11 (skin flaps)**

| **Variables** | **Rhythmic-*p*** | **Mesor** | **Amplitude** | **Acrophase** |
| --- | --- | --- | --- | --- |
| **PBS** | ＜.001 | 0.45463 ± 0.02379 | 0.35341 ± 0.03569 | 2.218 |
| **EXO** | < .001 | 0.53236 ± 0.0423 | 0.52561 ± 0.06259 | 1.668 |
| **MEXO** | < .001 | 0.73232 ± 0.06697 | 0.76115 ± 0.08119 | 2.730 |
| **SR-PBS** | < .001 | 0.39227 ± 0.01764 | 0.34987 ± 0.02304 | 11.721 |
| **SR-EXO** | < .001 | 0.51586 ± 0.02818 | 0.46237 ± 0.02915 | 9.324 |
| **SR-MEXO** | < .001 | 0.65602 ± 0.04513 | 0.67732 ± 0.05787 | 4.320 |

**Supplementary Figure 2: GPX4 (skin flaps)**

| **Variables** | **Rhythmic-*p*** | **Mesor** | **Amplitude** | **Acrophase** |
| --- | --- | --- | --- | --- |
| **PBS** | ＜.001 | 0.44944 ± 0.03172 | 0.3977 ± 0.04596 | 1.295 |
| **EXO** | < .001 | 0.51869 ± 0.04433 | 0.43374 ± 0.06676 | 2.300 |
| **MEXO** | < .001 | 0.63532 ± 0.06728 | 0.61808 ± 0.0996 | 1.686 |
| **SR-PBS** | < .001 | 0.36889 ± 0.01947 | 0.33112 ± 0.02798 | 12.932 |
| **SR-EXO** | < .001 | 0.50634 ± 0.02697 | 0.43802 ± 0.02997 | 9.941 |
| **SR-MEXO** | < .001 | 0.59081 ± 0.03067 | 0.55326 ± 0.04554 | 4.108 |

**Supplementary Figure 2: FTH1 (skin flaps)**

| **Variables** | **Rhythmic-*p*** | **Mesor** | **Amplitude** | **Acrophase** |
| --- | --- | --- | --- | --- |
| **PBS** | ＜.001 | 0.45554 ± 0.03429 | 0.41751 ± 0.05128 | 2.192 |
| **EXO** | < .001 | 0.5701 ± 0.06316 | 0.44346 ± 0.09541 | 3.019 |
| **MEXO** | < .001 | 0.75602 ± 0.05993 | 0.66694 ± 0.08713 | 1.254 |
| **SR-PBS** | < .001 | 0.38515 ± 0.02133 | 0.30076 ± 0.0319 | 14.179 |
| **SR-EXO** | < .001 | 0.5274 ± 0.03679 | 0.4696 ± 0.03338 | 9.823 |
| **SR-MEXO** | < .001 | 0.68643 ± 0.06099 | 0.48895 ± 0.0908 | 3.944 |

**Supplementary Figure 5: SLC7A11 (skin flaps)**

| **Variables** | **Rhythmic-*p*** | **Mesor** | **Amplitude** | **Acrophase** |
| --- | --- | --- | --- | --- |
| **shCtrl** | ＜.001 | 0.61816 ± 0.02888 | 0.47269 ± 0.04232 | 1.7227 |
| **SR-shCtrl** | ＜.001 | 0.48106 ± 0.02545 | 0.35744 ± 0.03644 | 12.8478 |
| **shARNTL** | ＜.001 | 0.46363 ± 0.02116 | 0.4226 ± 0.03118 | 13.6309 |

**Supplementary Figure 5: GPX4 (skin flaps)**

| **Variables** | **Rhythmic-*p*** | **Mesor** | **Amplitude** | **Acrophase** |
| --- | --- | --- | --- | --- |
| **shCtrl** | ＜.001 | 0.65443 ± 0.04965 | 0.50788 ± 0.07188 | 1.2506 |
| **SR-shCtrl** | ＜.001 | 0.38418 ± 0.02831 | 0.325 ± 0.04236 | 14.2075 |
| **shARNTL** | ＜.001 | 0.36549 ± 0.02456 | 0.32772 ± 0.03658 | 13.9805 |

**Supplementary Figure 5: FTH1 (skin flaps)**

| **Variables** | **Rhythmic-*p*** | **Mesor** | **Amplitude** | **Acrophase** |
| --- | --- | --- | --- | --- |
| **shCtrl** | ＜.001 | 0.5192 ± 0.01594 | 0.33773 ± 0.02203 | 0.5574 |
| **SR-shCtrl** | ＜.001 | 0.33611 ± 0.0215 | 0.28261 ± 0.02975 | 12.8851 |
| **shARNTL** | ＜.001 | 0.33594 ± 0.0152 | 0.3204 ± 0.02096 | 12.6431 |

**Supplementary Figure legends**

**Supplementary Fig. 1** **Inhibition of ferroptosis most effectively ameliorates flap necrosis induced by sleep restriction.** (A, D) Representative digital images and quantification of flap survival area on day 7 post-surgery (n = 3 mice / group). (B, E) Subcutaneous blood flow was detected by LDBF, the intensity signal of blood flow was analyzed (n = 3 mice / group). (C, F) Skin temperature was monitored using IRT, the flap temperature was analyzed (n = 3 mice / group). (G, H) The expression of CD34 (red) / USP4 (green) in flap of mice was analyzed by IF (n = 3 mice / group, scale bar = 100 μm). (I) Representative HE images of mice heart, liver, and kidney on day 7 (n = 3 mice / group, scale bars = 100 μm). One-way ANOVA was used for analysis that involved more than two groups; bar graph shows the mean ± SEM; **p* < .05, ***p* < .01 and ****p* < .001, ns = not signiﬁcant.

**Supplementary Fig. 2 MEXOs inhibit sleep restriction-induced ferroptosis in skin flap in mice.** (A) Serum corticosterone levels were measured across the indicated experimental groups (n = 18 mice / group). (B, D) WB was used to detect the expression of ferroptosis-associated proteins in flap on day 7 after SR (n = 3 mice / group). (C, E) WB was used to detect the expression of ferroptosis-associated proteins in flap with ferrostain-1 treatment (n = 3 mice / group). (F, H) The expression of CD34 (red) / USP4 (green) in flap of patients was analyzed by IF (n = 3 patients / group, scale bar= 100 μm). (G, I) The expression of CD34 (red) / USP4 (green) in flap was analyzed by IF (n = 3 mice / group, scale bar= 100 μm). (J-N) Circadian rhythm patterns for the 24h cycle of Corticosterone, SLC7A11, GPX4 and FTH1 using an ELISA assay (n = 3 mice / group). One-way ANOVA was used for analysis that involved more than two groups; bar graph shows the mean ± SEM; **p* < .05, ***p* < .01 and ****p* < .001, ns = not signiﬁcant.

**Supplementary Figure 3. Engineered BMSC-derived exosomes deliver functional USP4 to recipient cells *in vitro* and *in vivo*.** (A, B) IF staining of HUVECs following treatment with MEXOs [Flag-USP4] or MEXOs [Flag-Vec]. The Flag signal (red) co-localized with endogenous USP4 (green), demonstrating the delivery of exogenous Flag-USP4 (n = 3 / group, scale bars = 50 μm). (C, G) WB analysis of HUVECs confirming MEXOs [Flag-USP4] group exhibited higher total USP4 levels compared to those treated with MEXOs [Flag-Vec] (n = 3 / group). (D, H) WB analysis of flap tissues lysates confirming MEXOs [Flag-USP4] group exhibited higher total USP4 levels compared to those treated with MEXOs [Flag-Vec] (n = 3 mice / group). (E, F) Co-IP of HUVECs (n = 3 / group) and flap tissues lysates (n = 3 mice / group) using an anti-Flag antibody, followed by WB with an anti-USP4 antibody and anti-ARNTL, confirming an interaction between the delivered Flag-USP4 and endogenous ARNTL in recipient cells treated with MEXOs [Flag-USP4]. (I, J) A distinct Flag signal (red) was detected within vascular endothelial cells (marked by CD34, white) and co-localized with endogenous USP4 (green), providing visual proof of the incorporation of exosome-delivered Flag-USP4 into target cells *in vivo* (n = 3 mice / group, scale bars = 100 μm). Student’s t test was performed to compare two groups; bar graph shows the mean ± SEM; **p* < .05, ***p* < .01 and ****p* < .001, ns = not signiﬁcant.

**Supplementary Fig. 4 MEXOs inhibit ferroptosis by shuttling USP4, protecting the function of HUVECs.** (A, B) Cell migration assays were performed on HUVECs after 24 h of different treatments, and the presented results were obtained after 24h of culture in chambers, quantification and analysis of the number of migrated cells (n = 6 / group, scale bars = 100 μm). (C, D) Scratching assays were performed on HUVECs, and the presented results were obtained after 0h and 24h of culture, quantification and analysis of the migrated area of cells (n = 6 / group, scale bars = 100 μm). (E, F) An *in* *vitro* angiogenesis (tube formation) assay was performed on HUVECs, and the presented results were obtained after 4h of culture (n = 6 / group, scale bars = 100 μm). (G, H) The EdU assay showed the proliferation of HUVECs (n=6 / group, scale bars = 100 μm). (I, J) Detection and quantification of ROS levels in HUVECs by oxidation-sensitive fluorescent probe DCFH-DA (green, n = 6 / group, scale bars = 100 μm). (K, L) Detection and quantification of ROS levels in HUVECs by DHE staining (red, n = 6 / group, scale bars = 100 μm). (M-P) The level of apoptosis and ROS production was detected by flow cytometric analysis (n = 3 / group). (Q, R) WB was used to detect the expression of ferroptosis-associated proteins in HUVECs (n = 4 / group). (S, T) The expression of USP4 (red) in HUVECs was analyzed by IF with Erastin and ferrostain-1 treatment (n = 3 / group, scale bar= 100 μm). (U) The relative values of GSH, MDA, and Fe^2+^ concentrations in HUVECs with Erastin and ferrostain-1 treatment (n = 3 / group). One-way and two-way ANOVA were used for analysis that involved more than two groups; bar graph shows the mean ± SEM; **p* < .05, ***p* < .01 and ****p* < .001, ns = not signiﬁcant.

**Supplementary Fig.5 Disruption of the core clock gene ARNTL is sufficient to induce clock rhythmic ferroptosis and flap necrosis.** (A, B) Representative digital images and quantification of flap survival area on day 7 post-surgery (n = 3 mice / group). (C, D) Subcutaneous blood flow was detected by LDBF, the intensity signal of blood flow was analyzed (n = 3 mice / group). (E, F) Skin temperature was monitored using IRT, the flap temperature was analyzed (n = 3 mice / group). (G-I) Biochemical measurements of GSH, MDA, and Fe²⁺ levels in flap tissues (n = 3 mice / group). (J-L) Circadian rhythm patterns for the 24h cycle of ferroptosis-related proteins (n = 3 mice / group). *P* < 0.05 calculated by the Circa Compare algorithm was considered a circadian rhythm. The curve indicates the best fit to the points by cosinor analysis. R^2^ value represents the degree of the fitting. One-way ANOVA was used for analysis that involved more than two groups; bar graph shows the mean ± SEM; **p* < .05, ***p* < .01 and ****p* < .001, ns = not signiﬁcant.

**Supplementary Fig. 6 Exosomes containing USP4 inhibit ferroptosis by stabilizing ARNTL, protecting the function of HUVECs.** (A, B) Cell migration assays were performed on HUVECs after 24 h of different treatments, and the presented results were obtained after 24h of culture in chambers, quantification and analysis of the number of migrated cells (n = 3 / group, scale bars = 100 μm). (C, D) Scratching assays were performed on HUVECs, and the presented results were obtained after 0h and 24h of culture, quantification and analysis of the migrated area of cells (n = 3 / group, scale bars = 100 μm). (E, F) An *in vitro* angiogenesis (tube formation) assay was performed on HUVECs, and the presented results were obtained after 4h of culture (n = 3 / group, scale bars = 100 μm). (G, H) The EdU assay showed the proliferation of HUVECs (n=3 / group, scale bars = 100 μm). (I, K) Detection and quantification of ROS levels in HUVECs by oxidation-sensitive fluorescent probe DCFH-DA (green, n = 3 / group, scale bars = 100 μm). (J, L) Detection and quantification of ROS levels in HUVECs by DHE staining (red, n = 3 / group, scale bars = 100 μm). (M-P) The level of apoptosis and ROS production was detected by flow cytometric analysis (n = 3 / group). (Q, R) WB was used to detect the expression of ferroptosis-associated proteins in HUVECs (n = 3 / group). One-way ANOVA was used for analysis that involved more than two groups; bar graph shows the mean ± SEM; **p* < .05, ***p* < .01 and ****p* < .001, ns = not signiﬁcant.

**Supplementary Fig. 7 The m6A reader protein YTHDF1 interacted with USP4 mRNA and was found to be increased in BMSCs treated with melatonin.** (A) RNA dot blot analysis of m6A levels in BMSCs treated with MT. Methylene blue staining served as a loading control (n = 4 / group). (B) The total m6A levels in BMSCs were measured by EpiQuik M6A RNA Methylation Quantification Kit (n = 4 / group). (C) MeRIP- qPCR analysis of m6A levels of USP4 mRNA in BMSCs treated with MT (n = 4 / group). (D, E) RIP-qPCR and RNA affinity-isolation analysis of the interaction between YTHDF1 or METTL3 and USP4 mRNA in BMSCs treated with MT (n = 4 / group). (F) qPCR analysis of USP4, YTHDF1 and METTL3 mRNA expression levels in BMSCs treated with MT (n = 3 / group). (G, H) WB analysis of USP4, YTHDF1 and METTL3 expression levels in BMSCs (n = 3 / group). (I) qPCR analysis of USP4, YTHDF1 and METTL3 mRNA expression levels in exosomes (n = 3 / group). (J, K) WB analysis of USP4, YTHDF1 and METTL3 expression levels in exosomes (n = 3 / group). Student’s t test was performed to compare two groups, one-way and two-way ANOVA were used for analysis that involved more than two groups; bar graph shows the mean ± SEM; **p* < .05, ***p* < .01 and ****p* < .001, ns = not signiﬁcant.

**Supplementary Fig 1**

**
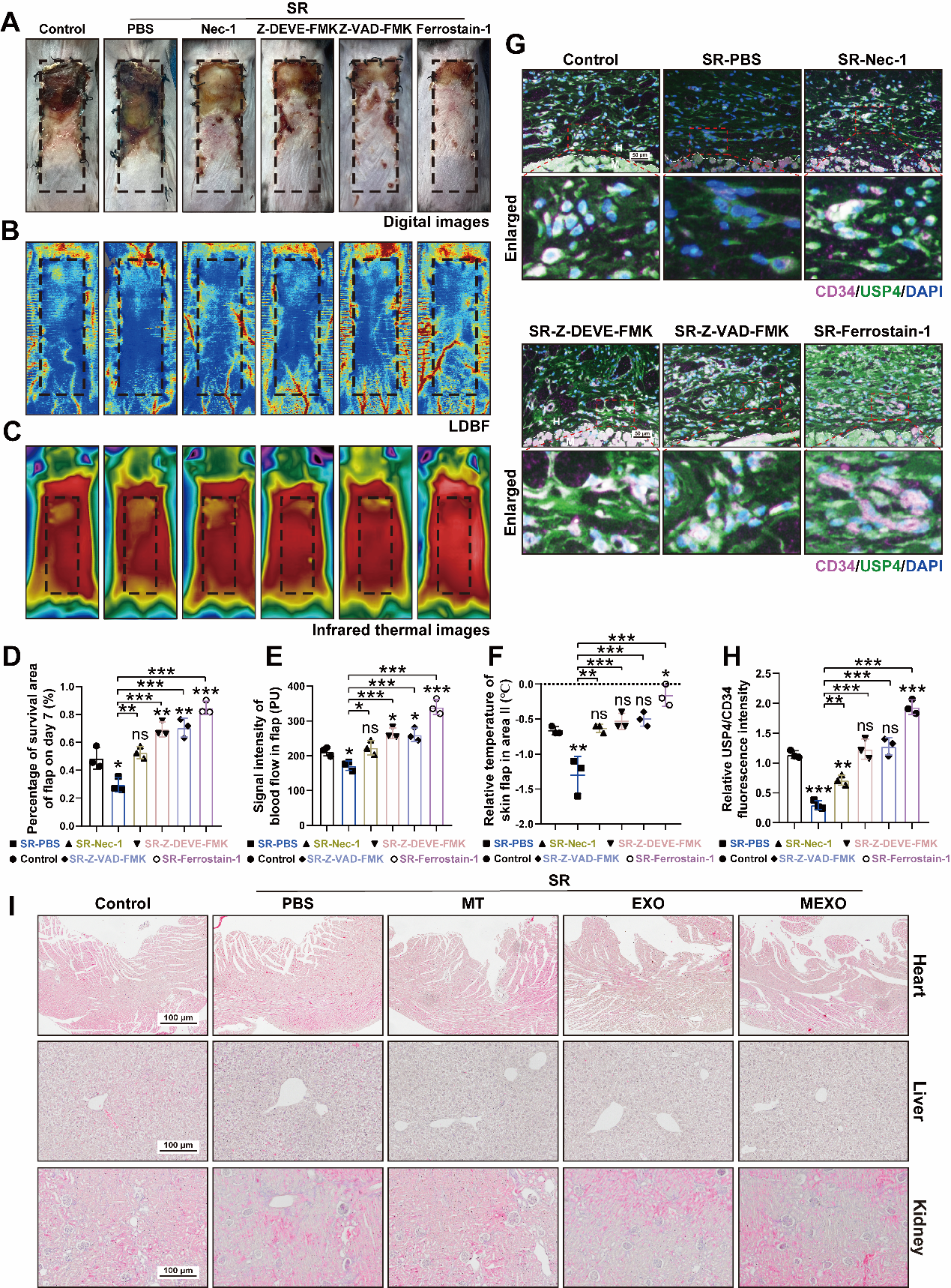
**

**Supplementary Fig 2**

**
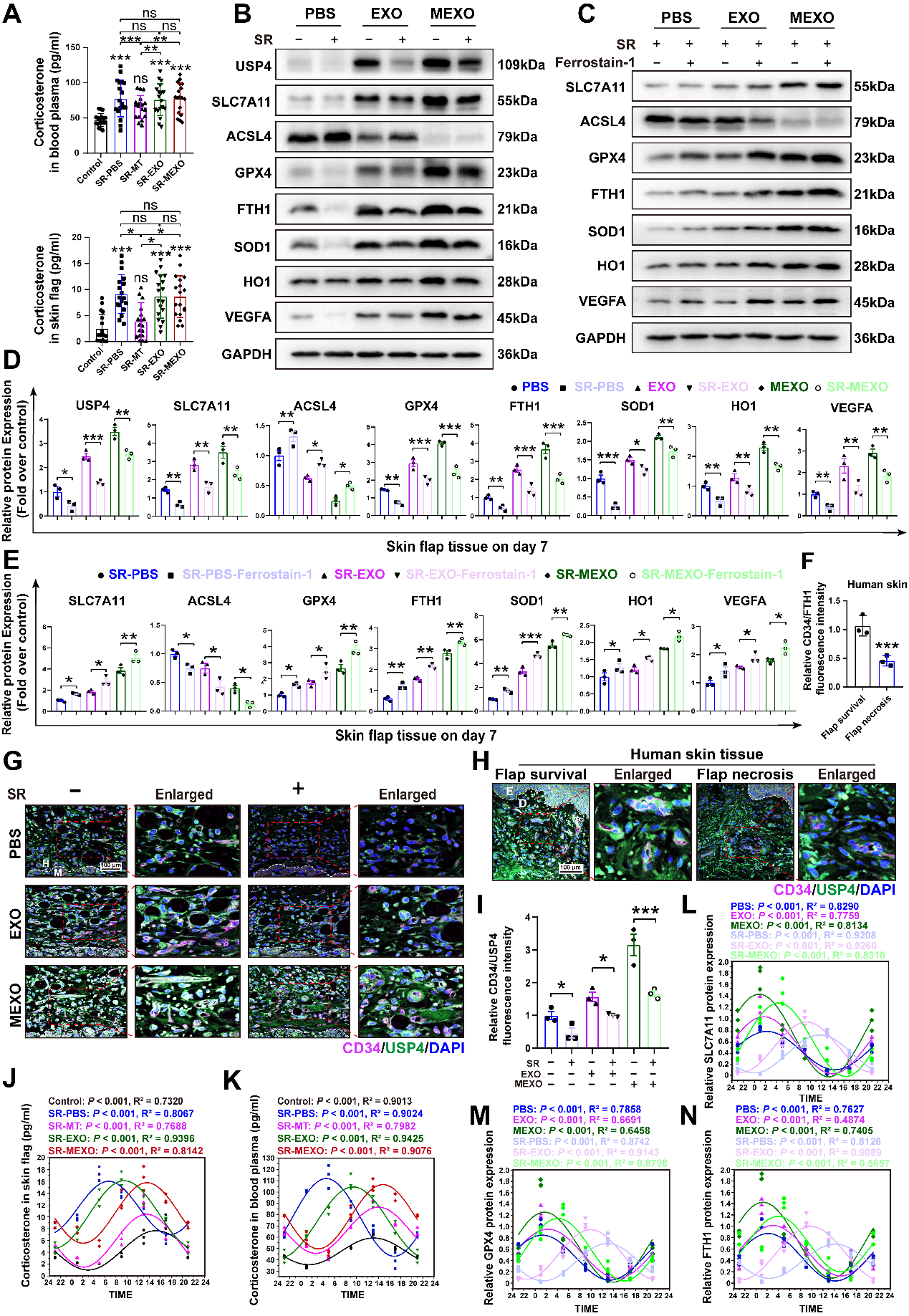
**

**Supplementary Fig 3**

**
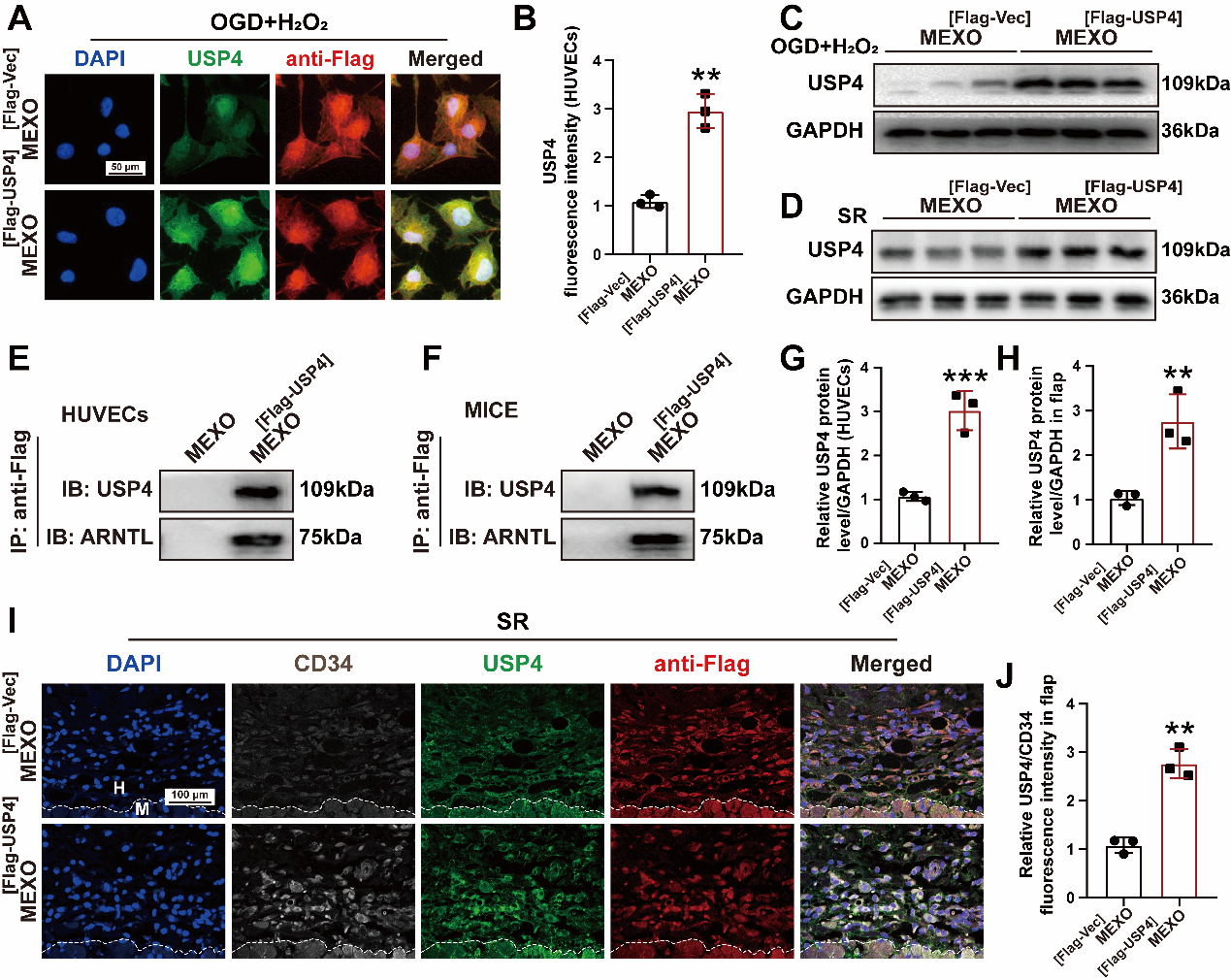
**

**Supplementary Fig 4**

**
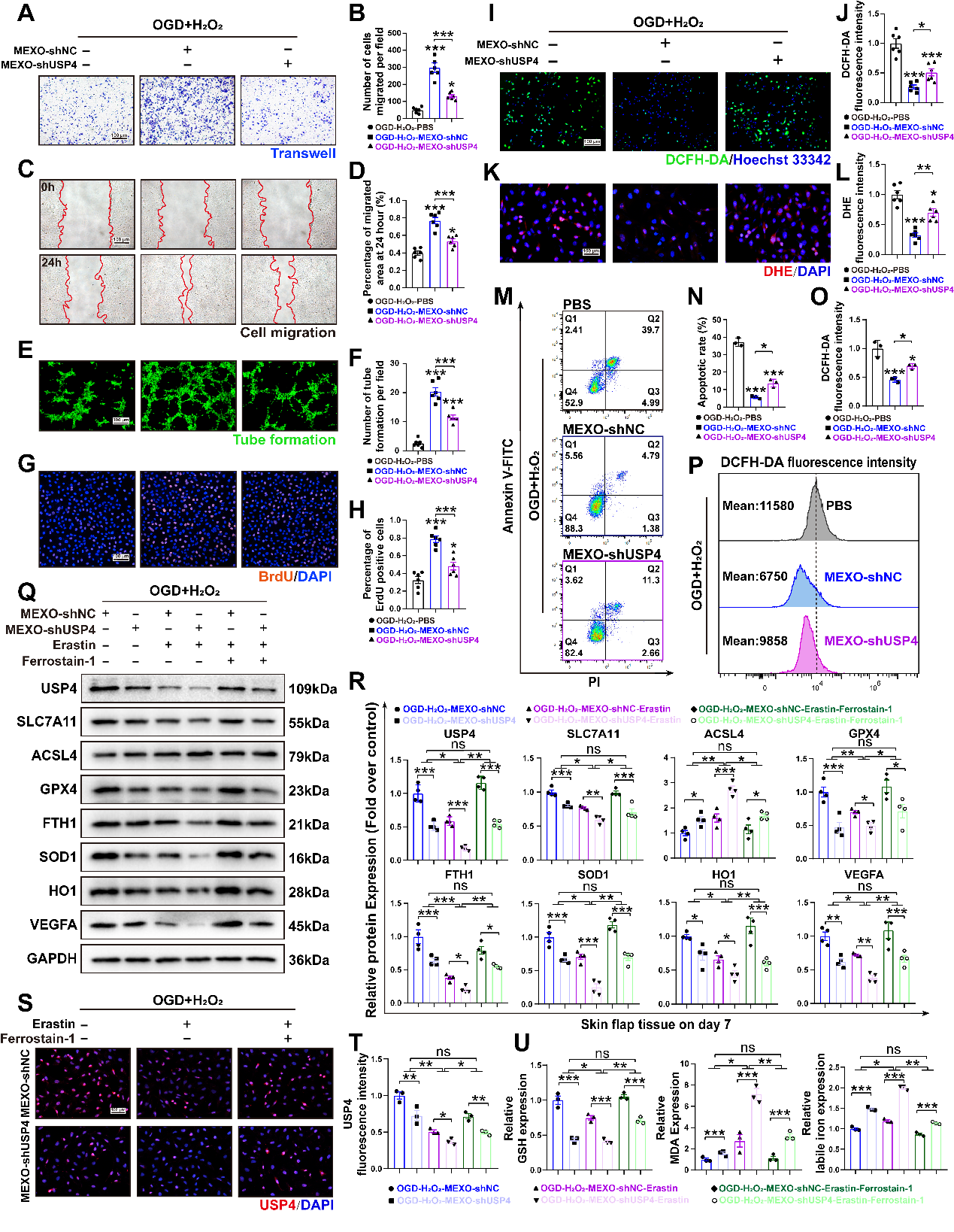
**

**Supplementary Fig 5**

**
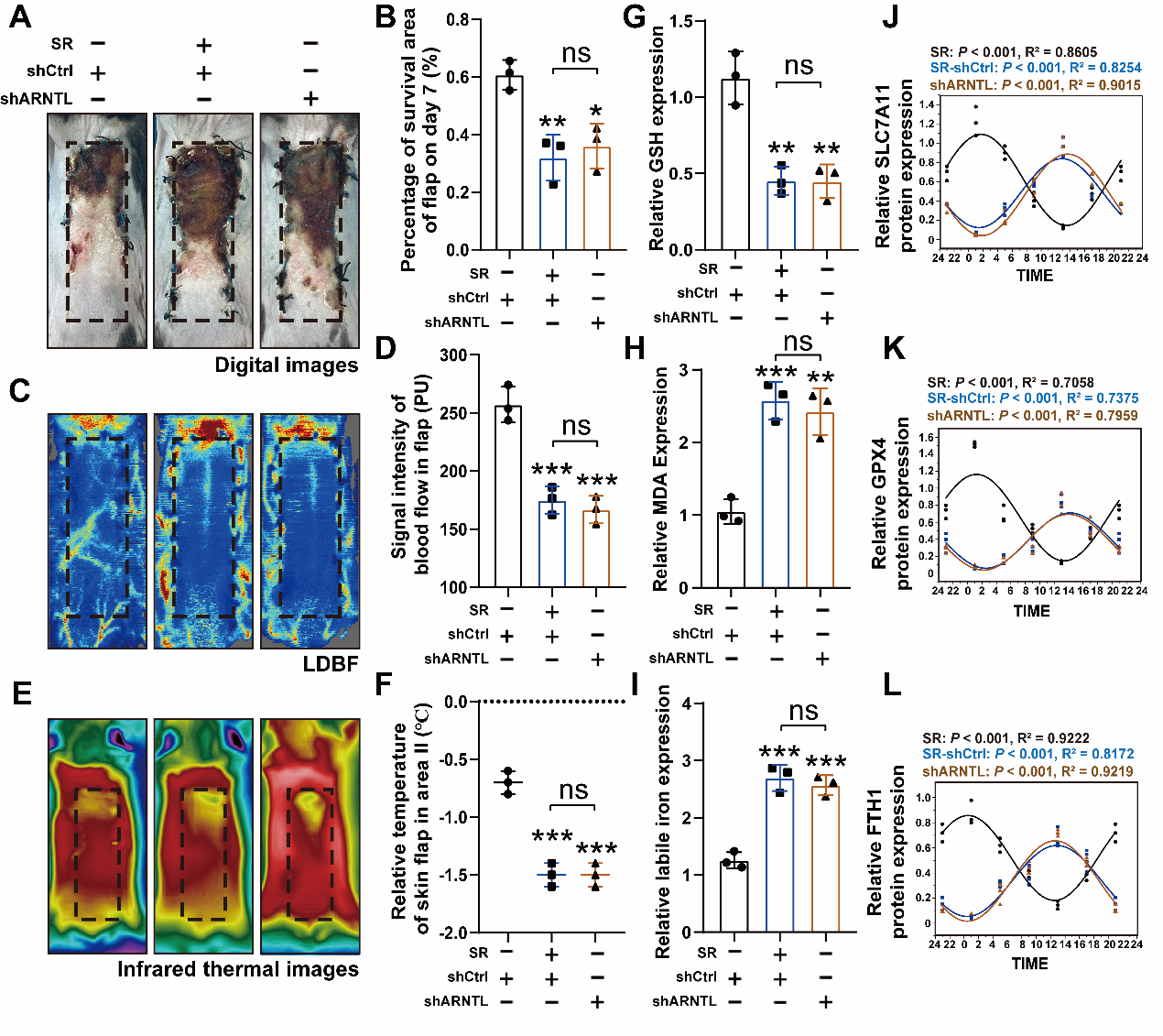
**

**Supplementary Fig 6**

**
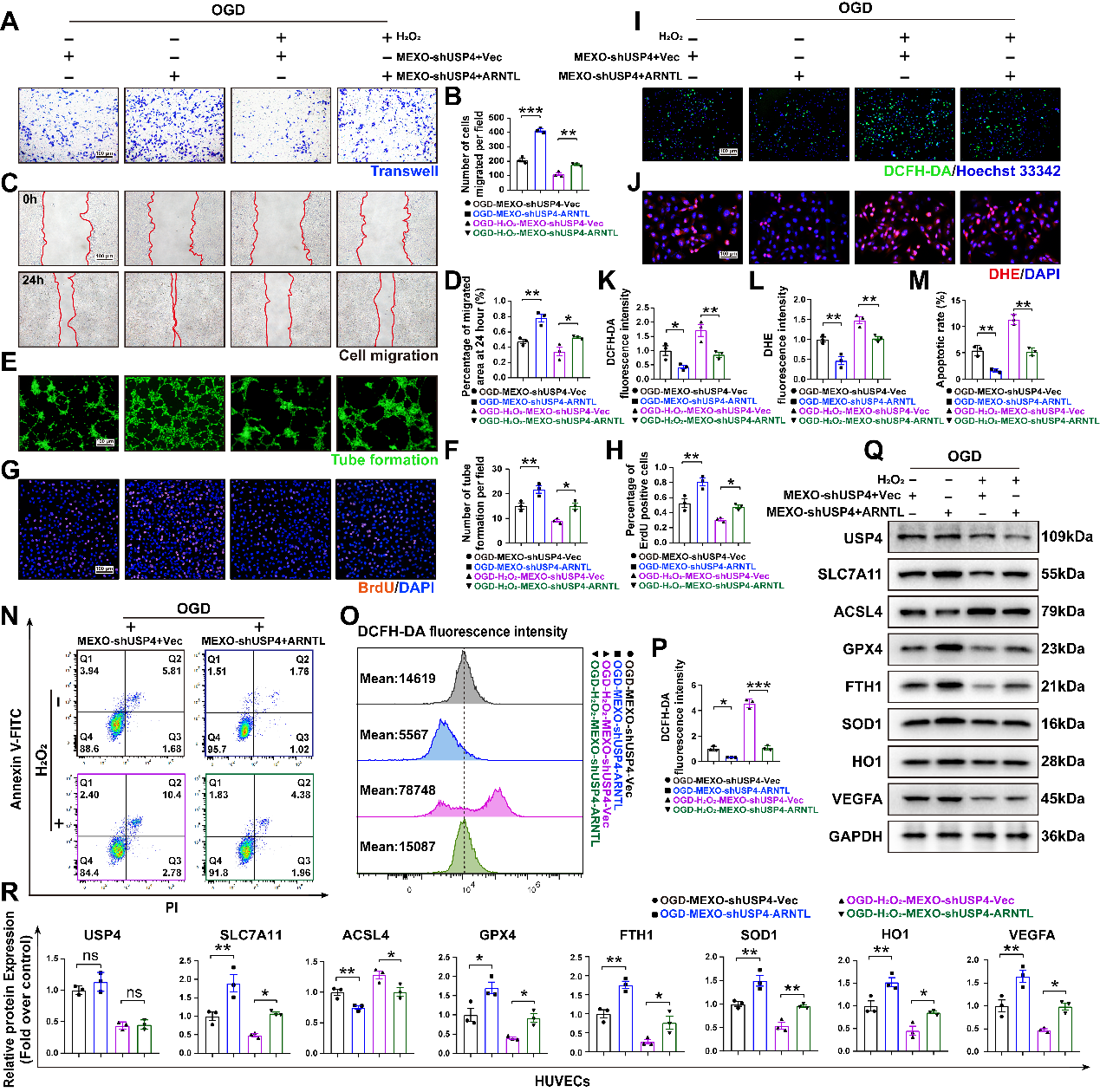
**

**Supplementary Fig 7**

**
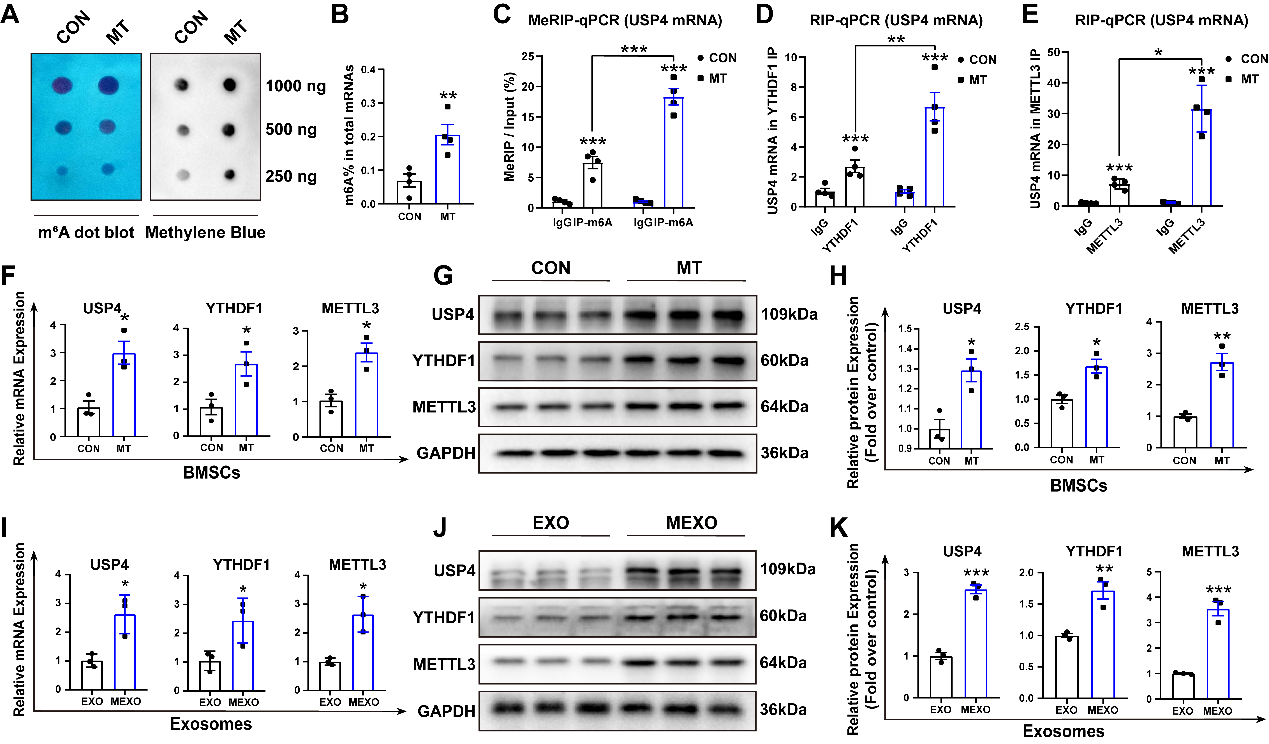
**
